# Dark Septate Endophytes Isolated From Wild Licorice Roots Grown in the Desert Regions of Northwest China Enhance the Growth of Host Plants Under Water Deficit Stress

**DOI:** 10.3389/fmicb.2021.522449

**Published:** 2021-06-23

**Authors:** Chao He, Wenquan Wang, Junling Hou, Xianen Li

**Affiliations:** ^1^Institute of Medicinal Plant Development, Chinese Academy of Medical Sciences, Peking Union Medical College, Beijing, China; ^2^School of Chinese Pharmacy, Beijing University of Chinese Medicine, Beijing, China

**Keywords:** licorice, dark septate endophytes, desert ecosystem, drought stress, inoculation

## Abstract

This study aimed to explore dark septate endophytes (DSE) that may improve the cultivation of medicinal plants in arid ecosystems. We isolated and identified eight DSE species (*Acremonium nepalense*, *Acrocalymma vagum*, *Alternaria chartarum*, *Alternaria chlamydospora*, *Alternaria longissima*, *Darksidea alpha*, *Paraphoma chrysanthemicola*, and *Preussia terricola*) colonizing the roots of wild licorice (*Glycyrrhiza uralensis*) in the desert areas of northwest China. Moreover, we investigated the osmotic stress tolerance of the DSE using pure culture, along with the performance of licorice plants inoculated with the DSE under drought stress in a growth chamber, respectively. Here, five species were first reported in desert habitats. The osmotic-stress tolerance of DSE species was highly variable, *A. chlamydospora* and *P. terricola* increased the total biomass and root biomass of the host plant. All DSE except *A. vagum* and *P. chrysanthemicola* increased the glycyrrhizic acid content; all DSE except *A. chartarum* increased the glycyrrhizin content under drought stress. DSE × watering regimen improved the glycyrrhizic acid content, soil organic matter, and available nitrogen. Structural equation model analysis showed that DSE × watering regimen positively affected soil organic matter, and total biomass, root length, glycyrrhizic acid, and glycyrrhizin (Shapotou site); and positively affected soil organic matter, available phosphorus, and glycyrrhizin (Minqin site); and positively affected the root length (Anxi site). DSE from the Shapotou site accounted for 8.0, 13.0, and 11.3% of the variations in total biomass, root biomass, and active ingredient content; DSE from the Minqin site accounted for 6.6 and 8.3% of the variations in total biomass and root biomass; DSE from the Anxi site accounted for 4.2 and 10.7% of the variations in total biomass and root biomass. DSE × watering regimen displayed a general synergistic effect on plant growth and active ingredient contents. These findings suggested that the DSE–plant interactions were affected by both DSE species and DSE originating habitats. As *A. chlamydospora* and *P. terricola* positively affected the total biomass, root biomass, and active ingredient content of host plants under drought stress, they may have important uses as promoters for the cultivation of licorice in dryland agriculture.

## Introduction

Licorice (*Glycyrrhiza uralensis* Fisch.) belongs to the Leguminosae family, and it has been widely used in the medicine and food processing industries (Michielsen et al., 2018). In China, licorice plants are mainly distributed in the northern regions. Licorice has been formally listed in Chinese Pharmacopeia due to its pharmacological ingredients such as glycyrrhizin and glycyrrhizic acid and associated biological functions (Kao et al., 2014; He et al., 2019a). In addition, licorice has been used to restore degraded soil in China because of its strong adaptability to low-fertility and arid soil (Chen et al., 2017). For decades, licorice cultivation in China has been used as a source of eco-economic value. However, the production and quality of cultivated licorice remain inadequate (Li et al., 2012).

The rhizosphere of plants usually contains diverse microorganisms, including those related to drought stress tolerance and plant growth promotion (Li et al., 2015; Lata et al., 2018; Suleiman et al., 2019). For example, rhizobia, symbiotic nitrogen-fixing bacteria, are widely found in arid soil and can significantly increase the plant biomass of *G. uralensis* (Hao et al., 2019). Fungal endophytes can enhance the tolerance of plants to drought stress, salt, and high temperature stress (Sarkar et al., 2015; Aghai et al., 2019; He et al., 2019b). These host-related endophytic fungi, such as arbuscular mycorrhizal fungi, *Trichoderma* spp., and dark septate endophytes (DSE), can be used as growth promoters or biofertilizers, in line with modern trends in crop management, environmental conservation, and functional food production (Acuna-Rodriguez et al., 2019; He et al., 2019a; Szczalba et al., 2019; Xie et al., 2019).

Dark septate endophytes can colonize the roots of mycorrhizal and non-mycorrhizal plants in a variety of extreme environments (Mandyam and Jumpponen, 2005; Hou et al., 2019). After successful colonization, typical dark septate hyphae and microsclerotia grow inter- and intra-cellularly in the plant root tissues (Xie et al., 2017). Moreover, the varied extensive characteristics of DSE in different habitats highlight their ecological roles, especially under stress environments, which are important for plant survival, biodiversity conservation, and ecosystem function stability (Pennisi, 2003; Lugo et al., 2015; Xu et al., 2015; Hou et al., 2019).

Although DSE species are commonly associated with plants throughout ecosystems, their germplasm resources and ecological roles need to be further clarified. At present, more than 600 plant species from 114 families associating with DSE colonizing their roots have been confirmed (Yuan et al., 2010; Tienaho et al., 2019). Novel DSE species and DSE-plant symbionts are constantly being discovered (Mahmoud and Narisawa, 2013; de Marins and Carrenho, 2017; Xie et al., 2017; Hou et al., 2019). The effects of DSE inoculation on plants range from positive to negative (Tellenbach et al., 2011; Li et al., 2019a). As an accelerator, DSE can promote plant growth by increasing nutrient and water absorption (Li et al., 2018; Yakti et al., 2018; He et al., 2019a), and by protecting plants from biotic and abiotic stresses (Likar and Regvar, 2013; dos Santos et al., 2017). Some studies have shown that inoculation with DSE can improve the production and quality of medicinal plants (Zhu et al., 2015; He et al., 2019a).

Water deficiency is one of the important abiotic factors that affect the sustainable development of agriculture (Lopes et al., 2011; Li et al., 2019b). Although host plants exhibit many adaptive strategies to mitigate or overcome the effects of stress, mycorrhiza are becoming a key highly effective approach to combat the effective ways of stress and thereby improve plant growth in arid environments (Gianinazzi et al., 2010; Li et al., 2019b). In fact, several studies have shown the importance of DSE inoculants in alleviating drought stress in plants (Nagabhyru et al., 2013; He et al., 2019b). Our previous study showed that inoculation of DSE species (*Acrocalymma vagum* and *Paraboeremia putaminum* from cultivated licorice plants in a farmland plot) improve licorice plant growth after sterilization treatment (He et al., 2019a).

The current study aimed to explore the diversity of DSE from wild licorice in the northwest deserts of China; to assess their osmotic stresses tolerance *in vitro*; and to evaluate the effects of DSE species from different desert habitats on the growth of licorice seedlings under drought conditions. First, DSE species were isolated and identified from the root of wild licorice in the desert area of northwest China. Second, the DSE species were exposed to low osmotic potential induced by polyethylene glycol (PEG) 6000, and their tolerance to osmotic stress was compared. Third, the effects of DSE on the growth performance of licorice plants under drought stress was studied. We mainly focused on the following questions: (1) What is the diversity of DSE species from wild licorice plants in northwest desert regions of China? (2) Do DSE species from desert habitats show high tolerance to osmotic stress *in vitro*? (3) Does inoculation of DSE improve the performance of licorice plants under drought stress? And (4) does drought stress affect the interaction between DSE and medicinal plants?

## Materials and Methods

### Study Sites and Sampling

Wild licorice plants were collected from the Shapotou Desert Research and Experiment Station of the Chinese Academy of Sciences (37°46′N, 105°01′E), Ningxia Province; Minqin Liangucheng National Nature Reserve (38°84′N, 103°27′E), and Anxi Extra-Arid Desert National Nature Reserve (40°43′N, 96°32′E), Gansu Province. These three areas are typical desert ecosystems in northwest China, with remarkable seasonal and diurnal temperature variations. Licorice is always a dominant species there. The mean annual precipitation at the Shapotou, Minqin, and Anxi sites is 186.6 mm, 113.9 mm, and 45.7 mm, respectively. The soils in the three sites are composed of Entisols and Aridisols (Soil Survey Staff, 2014). The soil physicochemical properties of the Shapotou, Minqin, and Anxi sites are as follows: pH: 8.15, 8.16, and 8.18; organic matter: 1.11, 0.91, and 2.05%; available nitrogen (N): 0.034, 0.037, and 0.027 g/kg; and available phosphorus (P): 0.006, 0.005, and 0.001 g/kg, respectively.

Three sample plots were chosen at each site in July 2018. Five replicate soil samples (at a depth of 30 cm) containing the fine roots of wild licorice were randomly selected from the rhizospheres of native wild licorice plants in each plot. The distance between the plants that were sampled was 100 m. We collected a total of 30 samples, stored them in sealed plastic bags, and transported them to the laboratory in an insulated container within 48 h. Before processing, the soil samples were sieved (2-mm mesh size) and fine roots were collected from each sample. Subsamples from each replicate were used to determine the physicochemical properties of the soils.

Mature seeds of licorice were collected from natural populations in Gansu Province and stored at 4°C.

### Quantification of Fungal Colonization

The roots were cut into 0.5-cm-long segments and stained with 0.5% (w/v) acid fuchsin after washing them under tap water and subjecting them to clearing using 10% (w/v) KOH (Phillips and Hayman, 1970). The glass slide method was used to assess fungal colonization status, with 20 random root segments being examined under microscope at 200× and 400× magnification (Biermann and Linderman, 1980). The value of colonization rate by DSE (hyphal, microsclerotial, and total) was calculated as the percentage of infected root segments in each root sample.

### Endophytic Fungal Morphological and Genetic Characterization

Root samples were surface-sterilized by sequential washes in 75% ethanol for 5 min and 10% NaClO for 5 min, followed by washing three times in deionized water. Finally, root segments were placed on potato dextrose agar (PDA) culture plates with ampicillin and streptomycin sulfate and cultured at 27°C with daily observation. Colonies with dark mycelium were isolated for further growth and fungal characterization, involving examination of the colony morphology and microscopic morphological traits were observed. Moreover, each isolate was cultured three times for 2 months at 10°C to induce sporulation (He et al., 2019a).

Fresh mycelia (approximately 50 mg) were scraped from the surface of PDA plates, and DNA was extracted using a genomic DNA extraction kit (Solarbio, China). Two primers, ITS4 (5′-TCCTCCGCTTATTGATATGC-3′) and ITS5 (5′-GGAA GTAAAAGT CGTAACAAGG-3′) were used for all isolates (Xie et al., 2017). PCR was conducted in a Life ECO^TM^ system (BIOER, China) in a reaction system (40 μL) that comprised 7 μL of DNA template, 1 μL of each primer, 20 μL of 2 × Es Taq Master Mix, and 11 μL of ddH_2_O. The PCR program was as follows: initial denaturation for 5 min at 94°C, 35 cycles of denaturation for 1 min at 94°C, primer for 1 min at 55°C, and extension for 1 min at 72°C, followed by final extension for 10 min at 72°C (He et al., 2019a). Finally, the PCR products were purified using Exonuclease I (20 U μL^–1^) and FastAP Thermosensitive Alkaline Phosphatase (1 U μL^–1^). Sequencing reactions were performed in a 3500 Series Genetic Analyzer using a BigDye^TM^ Terminator Cycle Sequencing Kit v.3.1 (Applied Biosystems^TM^). Sequences were stored in GenBank under accession numbers MN517851 (DSE1), MN517852 (DSE2), MN517853 (DSE3), MN517854 (DSE4), MN517855 (DSE5), MN517856 (DSE6), MN517857 (DSE7), and MN517858 (DSE8). Sequence alignment was completed using Clustal X (v.1.81), and MEGA 6 was used to construct a maximum likelihood phylogenetic tree (Tamura et al., 2013).

### Osmotic Stress Tolerance of DSE *in vitro*

Osmotic stress was brought about by adding PEG 6000 to pure modified Melin–Norkrans (MMN) medium (pH 5.5) (Hosseini et al., 2019) to achieve osmotic potentials of 0, –0.45, –1.34, and –1.79 MPa (Chen et al., 2003). A 5-mm disc of inoculum was cut from each isolate and placed in a 250-mL Erlenmeyer flask with 100 mL of liquid MMN. The flasks were cultured for 10 days in the dark with continuous shaking. The fungal mycelium was rinsed with distilled water several times before harvesting. There were five replicates for each osmotic stress level. Superoxide dismutase (SOD) activity, and malondialdehyde (MDA) and melanin content were directly determined using a portion of each fungal sample from each replicate. Thereafter, the other portions were weighed before drying to constant weight at 80°C. The biomass of each repeated culture was the sum of the dry weight of two parts.

### Superoxide Dismutase Activity and MDA Content Determination

Fresh hyphae (0.2 g) were homogenized in 5 mL of reaction mixture (extracted in 50 mM phosphate buffer with 0.2 mM EDTA, and 2% polyvinylpyrrolidone); pH 7.8 at 4°C in a precooled mortar. The homogenate was centrifuged for 30 min at 15000 × *g* and 4°C, and then the supernatant was collected for SOD activity analysis. The SOD activity was determined using the photochemical method described by Elavarthi and Martin (2010), based on the decrease in the absorbance by a nitroblue tetrazolium (NBT) complex, which is related to SOD activity. One unit of SOD activity indicates the quantity of enzyme needed to inhibit NBT reduction by 50%, assessed based on absorbance at 560 nm.

The thiobarbituric acid (TBA) method was used to assess the MDA content (Peever and Higgins, 1989). Briefly, fresh hyphae (0.2 g) from each isolate were homogenized in 10% trichloroacetic acid (TCA; 5 mL), and then centrifuged for 10 min at 12000 × *g*. Next, 2 mL of supernatant and 2 mL of 0.5% thiobarbituric acid (TBA) were mixed in a boiling water bath. After 15 min, the mixture was swiftly cooled and then centrifuged for 10 min at 12000 × *g*. Absorbance of the supernatant was measured using a spectrophotometer at 450, 532, and 600 nm. The MDA content was calculated using the following formula:

C (μmol/L) = 6.45(OD_532_–OD_600_)–0.56(OD_450_)

### Melanin Content Determination

To determine the melanin content in mycelia, an extraction protocol was carried out as described by Ellis and Griffiths (1974). Briefly, melanin was extracted from hyphae with hot alkali solution (1 M NaOH at 100°C) for 4 h in a water bath. The cooled cell extract was filtered through a double layer of filter paper and acidified with concentrated HCl (7 M) until precipitation at pH 2.0. The resulting dark brown precipitate was recovered by centrifugation at 10,000 × *g* for 15 min and washed with distilled water. The coagulated melanin was then dissolved in 1 M NaOH, and the yield of melanin was determined. The melanin content (expressed as mg/g) was measured using a standard curve showing absorbance at 459 nm.

### Dark Septate Endophytes Inoculation Experiment

The experiment was conducted in a growth chamber using a completely randomized factorial design (9 inoculation treatments × 2 watering regimes) with five replicates. A total of 90 pots were prepared. The inoculation treatments comprised DSE1, DSE2, DSE3, DSE4, DSE5, DSE6, DSE7, DSE8, and a non-inoculated control. The watering regimes involved well-watered (WW) and drought stress (DS) conditions.

Licorice seeds were surface-disinfected with 70% ethanol for 3 min and with 2.5% sodium hypochlorite for 10 min, and then thoroughly washed with sterile water. Following germination on water agar medium (containing 10 g/L agar) at 27°C, the seedlings were cultivated in sterile pots (13 cm diameter, 12 cm height, 2 seedlings/pot) containing an 800 g sterilized culture substrate. For the inoculation treatments, two 5-mm disks were cut from a 14-day-old PDA culture medium and placed at 1 cm below the plant roots (He et al., 2019a). For the non-inoculated controls, two 5-mm disks were cut from the PDA culture medium without fungi and placed in the pot. All the inoculation procedures were conducted on a clean bench. Each pot was cultured in a growth chamber for 90 days, with a 14 h/10 h light/dark photoperiod, at 27°C/22°C (day/night) and a mean relative air humidity of 60%.

One month after sowing, half of the seedlings (inoculated and control) were subjected to WW treatment (70% of the field water capacity), and the rest were subjected to DS treatment (30% of field water capacity; the DS treatment was based on the median value in the natural habitat of licorice plants in northwest China). A soil humidity recorder (L99-TWS-2; China) was used to assess soil moisture. Sterile distilled water was added daily to maintain the designated percentage of field water capacity based on regular weighing.

### Plant Biomass and Morphological Traits

Plant height and leaf number from each pot were recorded before plant shoots and roots were separately harvested. The root system was gently rinsed with tap water to remove adherent sandy soil. Fresh roots were randomly selected and cut into 0.5-cm segments for DSE colonization assessment as described above. Individual root sections were floated in approximately 1-cm-deep water in a plexiglass tray and scanned using a desktop scanner (EPSON Perfection V800 Photo; Suwa, Japan). Root morphological traits, comprising total root length, root surface area, and mean root diameter, were assessed using a WinRHIZO image analysis system (Regent Instruments, Quebec, QC, Canada). The root sections were collected after scanning. Fresh shoots were dried at 70°C for at least 48 h prior to weighing.

### Determination of Active Component Content

Dried roots from each pot were ground with a mortar and sieved with a 40-mesh sieve. An approximately 0.05 g sample was accurately weighed and subjected to extraction using 10 mL of 70% methanol in an ultrasonic bath for 30 min, then cooled to 25°C and filtered through a 0.45-μm filter. Next, 10 μL of filtrate was separated via high-performance liquid chromatography using a reverse-phase C_18_ symmetry column (4.6 × 250 mm, pore size: 5 μm; Waters Corp., Milford, MA, United States). Separation was carried out at 25°C using a gradient elution mode (Supplementary Table 1), where the mobile phase consisted of deionized water: phosphoric acid (100:0.05, v/v) and acetonitrile. The flow rate was 1.0 mL/min. The levels of the eluted compounds were determined at 237 nm using a 2998 PDA photodiode array detector (Waters Corp.). Standard substances (glycyrrhizic acid and glycyrrhizin) were purchased from China National Institutes for Food and Drug Control (Zhang et al., 2013; He et al., 2019a).

### Soil Parameters

Rhizospheric soil samples that strongly adhered to the roots were collected, sieved (2-mm sieve), and dried at room temperature. Each dried soil sample (0.2 g) was digested in 10 mL of a 10:1:2 mixture of perchloric acid (12.7 mol/L), sulfuric acid (18 mol/L), and water using a Mars 6 microwave digestion system (CEM Corporation, Matthews, NC, United States) until a clear liquid was obtained. Soil organic matter was measured as the percentage of organic carbon using dichromate oxidization in H_2_SO_4_ (Rowell, 1994). Available N and P were determined by the alkaline hydrolysis diffusion method (Rowell, 1994) and the chlorostannus-reduced molybdophosphoric acid blue color method (Olsen et al., 1954), respectively.

### Statistical Analysis

All statistical analyses were performed in SPSS 21.0 (SPSS Inc., Chicago, IL, United States). One-way analysis of variance (ANOVA) was performed to analyze the effects of osmotic stress on the biomass, SOD activity, and MDA and melanin content of each DSE species. Two-way ANOVA was performed to examine the effects of DSE inoculation, watering regime, and DSE × watering regimen on plant biomass, morphological traits, glycyrrhizic acid content, glycyrrhizin content, and soil parameters. The pairwise differences in means were analyzed by Duncan’s multiple range test. *P* < 0.05 indicated statistical significance. The values in the figures are the means of at least three replicates. The Mantel test and structural equation modeling (SEM) were used to test the effects of DSE species, watering regimen, and soil parameters on the growth and active component content of licorice plants using R-3.2.2 package ecodist (Goslee and Urban, 2007) and AMOS 21.0 (maximum likelihood). The effect sizes of DSE from different sites, watering regime, and soil variables on the total biomass, root biomass, and the active component content of host plants were estimated by variation partitioning.

## Results

### Characterization and Identification of DSE Species in Wild Licorice Roots

In the roots of the licorice plants, we observed dark septate hyphae, ranging from brown to dark brown, and microsclerotia structures (Supplementary Figure 1). Specifically, the hyphae invaded the epidermal cells, cortical cells, or vascular tissue. The microsclerotia were chainlike and formed conglomerates, and they colonized single or multiple cortical cells. The DSE colonization rates for licorice plants from Shapotou, Minqin, and Anxi were as follows: hyphae: 72.2%, 71.7%, and 40.5%; microsclerotia: 32.8%, 40.6%, and 3.3%; and total: 78.3%, 80.3%, and 43.3%, respectively.

**FIGURE 1:**
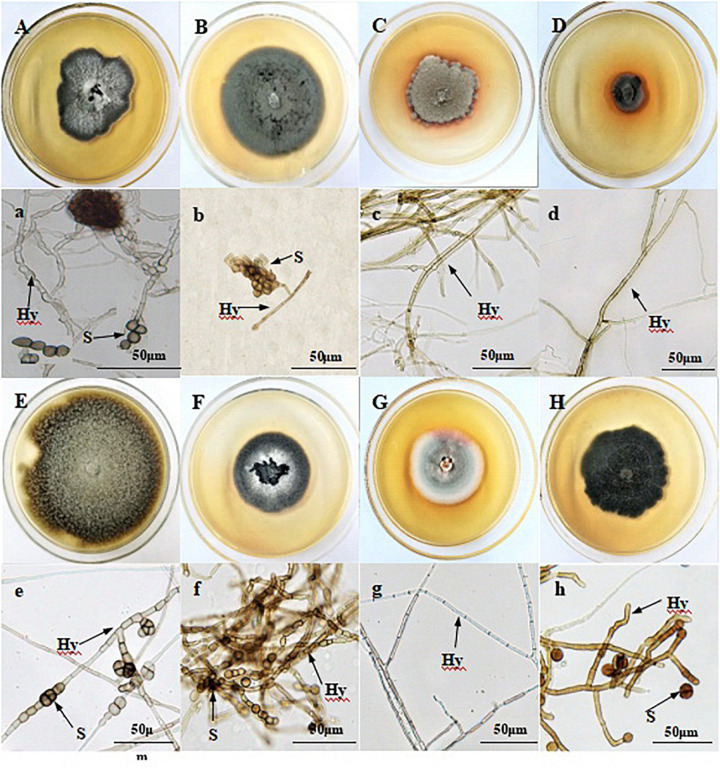
Colonies of endophytic fungi isolated from the roots of wild licorice **(A–H)**. Microscopic morphology of endophytic fungi **(a–h)** (Bars = 50 μm). **(A) a:** *Acrocalymma vagum* (DSE1); **(B) b:** *Paraphoma chrysanthemicola* (DSE2); **(C) c:** *Alternaria longissima* (DSE3); **(D) d:** *Darksidea alpha* (DSE4); **(E) e:** *Alternaria chlamydospora* (DSE5); **(F) f:** *Acremonium nepalense* (DSE6); **(G) g:** *Preussia terricola* (DSE7); **(H) h:** *Alternaria chartarum* (DSE8). Arrows indicate: Hy, DSE hyphae; S, DSE spores.

*In vitro*, DSE species varied in color from ashen to gray to dark brown (Figure 1). They had linear growth curves, and the mean growth rates of DSE1–8 were 0.29, 0.33, 0.21, 0.12, 0.29, 0.33, 0.21, and 0.26 cm/d, respectively. Although DSE1, DSE2, DSE5, DSE6, and DSE8 produced chlamydospores *in vitro*, neither conidia nor reproductive structures of other DSE species were observed. DSE1, DSE2, DSE3, and DSE4 were isolated in Sapotou; DSE5, DSE6, and DSE7 in Anxi; and DSE8 in Minqin.

### Molecular Phylogeny of DSE Species

**FIGURE 2:**
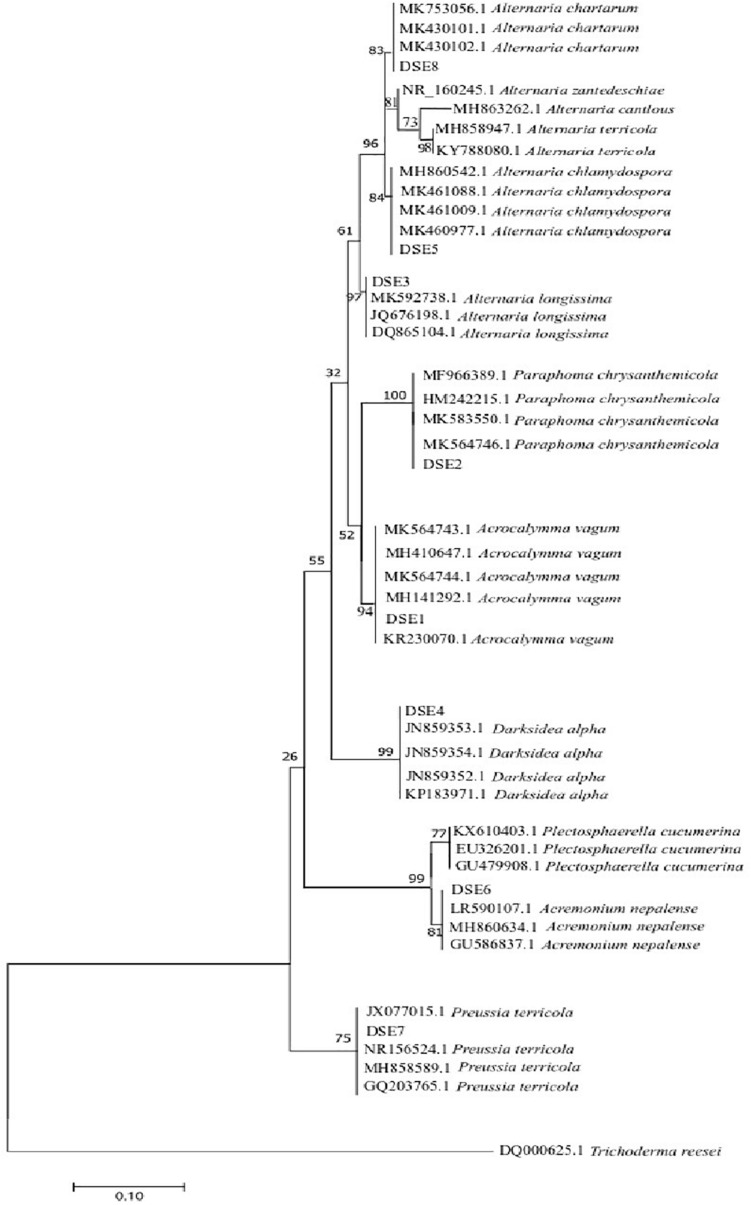
Maximum parsimony tree generated from ITS (ITS4 and ITS5) sequences of the isolate strains and their closest matches, followed by GenBank accession number.

A phylogenetic tree based on ITS4-5.8S-ITS5 rDNA is shown in Figure 2. According to the morphological characteristics and ITS sequence analysis, the DSE species were identified as *Acrocalymma vagum* (DSE1), *Paraphoma chrysanthemicola* (DSE2), *Alternaria longissima* (DSE3), *Darksidea alpha* (DSE4), *Alternaria chlamydospora* (DSE5), *Acremonium nepalense* (DSE6), *Preussia terricola* (DSE7), and *Alternaria chartarum* (DSE8).

### Dark Septate Endophytes Osmotic Stress Tolerance *in vitro*

**FIGURE 3:**
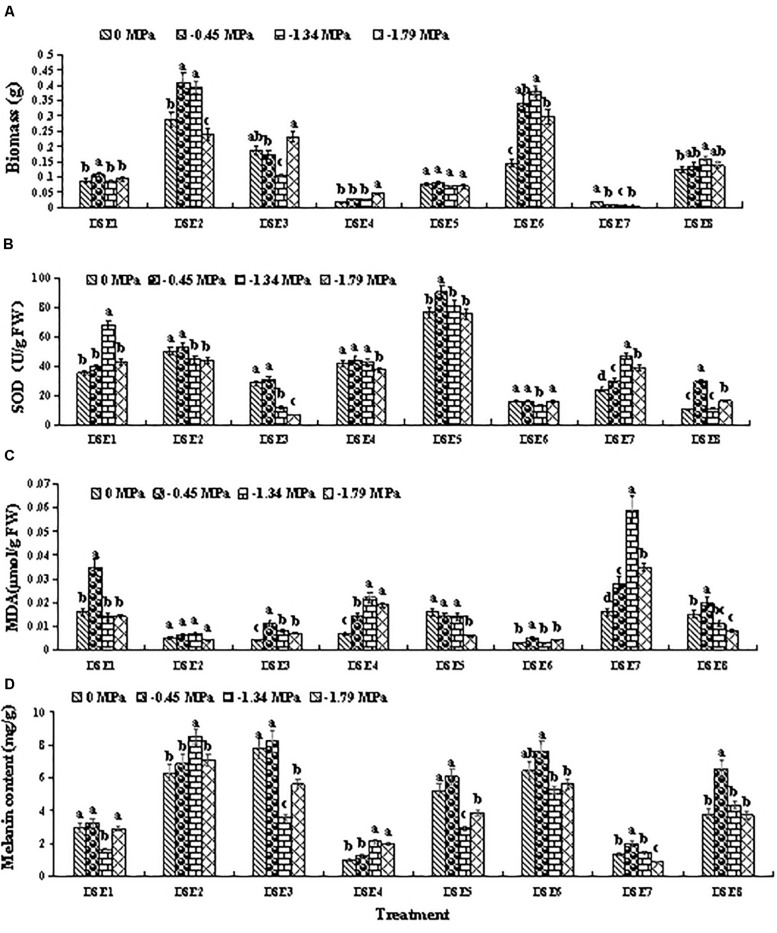
Response of the biomass **(A)**, superoxide dismutase (SOD) activity **(B)**, and malondialdehyde (MDA) **(C)**, and melanin content **(D)** in the eight dark septate endophytes (DSE) to different concentrations of polyethylene glycol (PEG) 6000. Different letters above the error bars indicate significant differences at *P* < 0.05.

All eight DSE species showed relatively high osmotic tolerance *in vitro* (Figure 3 and Table 1). However, the various DSE species exhibited different biomass growth (after 10 days of culturing) under osmotic stress relative to the unstressed condition (0 MPa). For instance, DSE6 exhibited higher biomass at –0.45, –1.34, and –1.79 MPa relative to the unstressed condition (0 MPa), whereas DSE7 exhibited lower biomass at these osmotic stress levels (Figure 3A).

**TABLE 1:** Analysis of variance (ANOVA) for the effects of dark septate endophytes and osmotic stress on the biomass, superoxide dismutase activity (SOD), and malondialdehyde (MDA), and melanin content of DSE species.

	Biomass (g)		SOD (U/g FW)		MDA (μmol/g FW)		Melanin (mg/g)	
	F	P	F	P	F	P	F	P
DSE	35.431	**<0.001**	232.357	**<0.001**	48.349	**<0.001**	73.775	**<0.001**
Osmotic stress	1.794	0.157	3.572	**0.019**	7.747	**<0.001**	12.767	**<0.001**
DSE × osmotic stress	1.617	**0.073**	10.308	**<0.001**	8.341	**<0.001**	4.134	**<0.001**

*Significant P-values are in bold.*

The various DSE species exhibited different SOD activity under osmotic stress relative to the unstressed condition (0 MPa). For instance, DSE5 exhibited higher SOD activity at –0.45 MPa, and DSE7 exhibited higher SOD activity at –0.45, –1.34, and –1.79 MPa, whereas DSE3 and DSE6 exhibited lower SOD activity at –1.34 MPa (Figure 3B).

The various DSE species showed different MDA content under osmotic stress relative to the unstressed condition (0 MPa). For example, DSE3, DSE4, and DSE7 exhibited higher MDA content at all osmotic stress levels, whereas DSE5 and DSE8 exhibited lower MDA content at –1.79 MPa (Figure 3C).

Lastly, the various DSE species showed different melanin content under osmotic stress relative to the unstressed condition (0 MPa). For example, DSE7 and DSE8 exhibited higher melanin content at –0.45 MPa, whereas DSE3 and DSE5 exhibited lower melanin content at –1.34 and –1.79 MPa (Figure 3D).

### Effects of DSE on Plant Morphological Parameters

**FIGURE 4:**
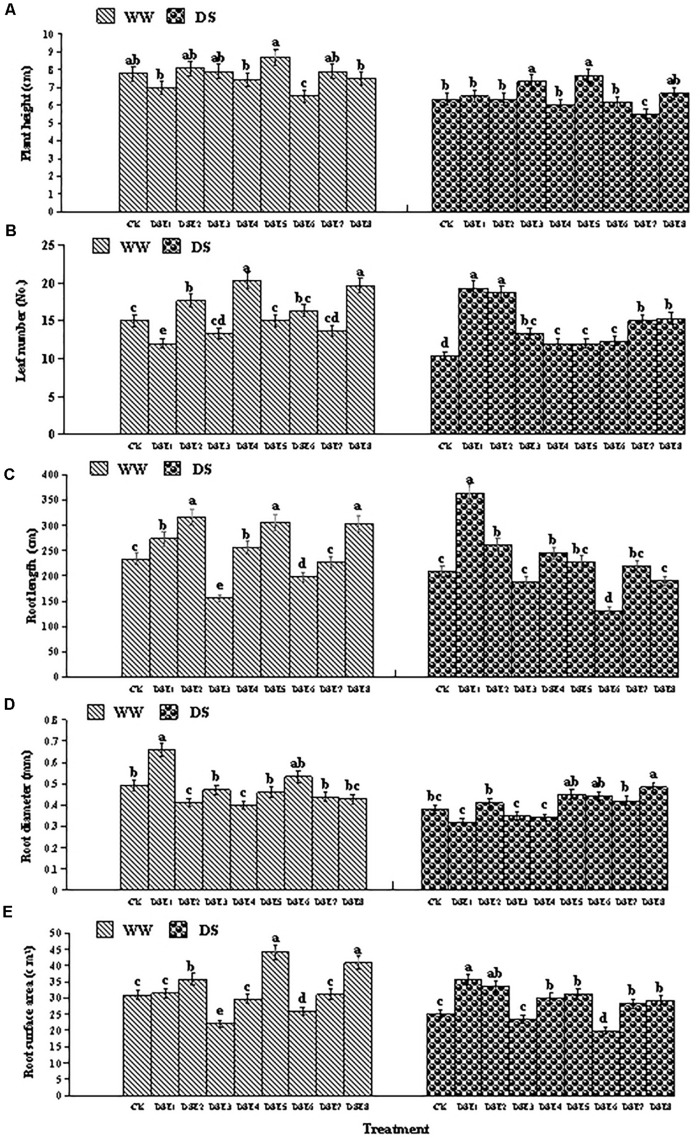
**(A–E)** Effects of dark septate endophytes (DSE) and water treatment on the morphological parameters of licorice plants. Different letters above the error bars indicate significant difference at *P* < 0.05. CK indicates non-inoculated plants. DSE1–DSE8, indicate plants inoculated with different DSE species. WW, DS, indicate well-watered and drought stress treatment, respectively.

All seedlings exhibited healthy growth throughout the experimental period. After harvesting, microscopic observation revealed that all DSE isolates successfully colonized the roots of licorice seedlings. The morphological parameters of the inoculated plants relative to the control plants are shown in Figure 4 and Table 2. In general, DSE significantly affected the height, leaf number, and root morphology of licorice plants, regardless of the watering regime.

**TABLE 2:** Two-way analysis of variance for the effects of dark septate endophytes (DSE) and water condition on the growth and active ingredient content of licorice plants.

	DSE		Water		DSE × water	
	F	P	F	P	F	P
Plant height (cm)	1.853	0.099	17.567	**<0.001**	0.69	0.697
Leaf number (No.)	5.363	**<0.001**	8.721	**0.006**	7.646	**<0.001**
Root length (cm)	4.436	**<0.001**	2.675	0.111	1.637	0.149
Root surface area (cm^2^)	2.284	**0.043**	2.851	0.1	0.677	0.708
Root diameter (cm)	2.648	**0.022**	24.049	**<0.001**	5.82	**<0.001**
Shoot biomass (g/pot)	1.235	0.307	24.454	**<0.001**	0.809	0.599
Root biomass (g/pot)	7.484	**<0.001**	99.743	**<0.001**	3.793	**0.003**
Total biomass (g/pot)	5.472	**<0.001**	86.486	**<0.001**	2.515	**0.028**
Root: shoot ratio	3.848	**0.002**	34.195	**<0.001**	3.156	**0.008**
Glycyrrhizic acid (%)	2.089	0.063	1.958	0.170	4.576	**<0.001**
Glycyrrhizin (%)	0.719	0.673	0.797	0.378	1.01	0.446

*Significant P-values are in bold.*

Under WW conditions, DSE6 reduced plant height relative to the control plants, whereas DSE3 and DSE5 increased it. Under DS conditions, DSE7 reduced plant height relative to the control plants.

Under WW conditions, DSE2, DSE4, and DSE8 increased the number of leaves relative to the control plants, whereas DSE1 reduced it. However, under DS conditions, all DSE increased the number of leaves relative to the control plants.

Regarding root morphology, DSE colonization affected the roots relative to control roots under both watering regimens. For instance, DSE1, DSE2, and DSE4 increased the root length, whereas DSE6 reduced it, regardless of the watering regime. Under WW conditions, DSE1 increased the root diameter, whereas DSE2 and DSE4 reduced it; under DS conditions, only DSE8 increased it. Under WW conditions, DSE2, DSE5, and DSE8 increased the root surface area, whereas DSE3 and DSE6 reduced it; under DS conditions, DSE1, DSE2, DSE4, DSE5, DSE7, and DSE8 increased it, whereas DSE6 reduced it (Figure 4). Notably, DSE × watering regimen significantly affected the leaf number and root diameter of host plants (Table 2).

### Effects of DSE on Plant Biomass

**FIGURE 5:**
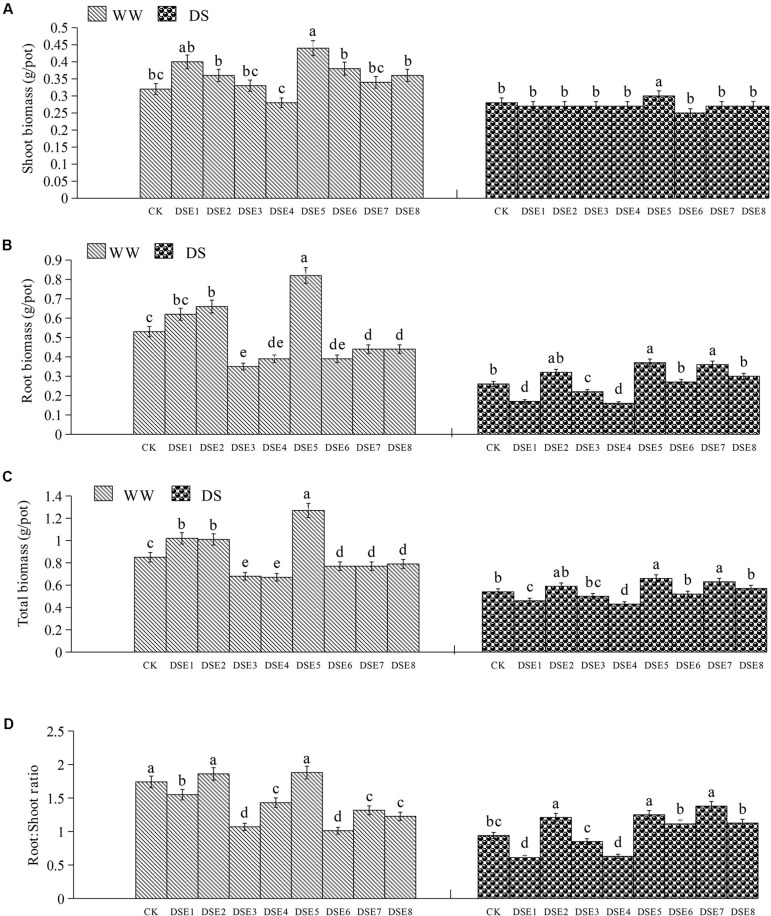
**(A–D)** Effects of dark septate endophytes (DSE) and water treatment on the biomass production of licorice plants. Different letters above the error bars indicate significant difference at *P* < 0.05. CK indicates non-inoculated plants. DSE1–DSE8, indicate plants inoculated with different DSE species. WW, DS, indicate well-watered and drought stress treatment, respectively.

Dark septate endophytes colonization significantly affected the biomass and root:shoot ratio of licorice plants. In particular, DSE5 increased the total, shoot, and root biomass relative to the control plants, whereas the other DSE species had no obvious effect on the shoot biomass, regardless of the watering regime. Under WW conditions, DSE1 increased the total biomass, and DSE2 increased the total and root biomass, whereas DSE3, DSE4, DSE6, DSE7, and DSE8 reduced these parameters, relative to the control plants. Under DS conditions, DSE7 increased the total and root biomass, whereas DSE1 and DSE4 reduced the total and root biomass, and DSE3 reduced the root biomass, relative to the control plants. Under WW conditions, DSE1, DSE3, DSE4, DSE6, DSE7, and DSE8 decreased the root:shoot ratio, whereas DSE1 and DSE4 decreased it; under DS conditions, DSE2, DSE5, and DSE7 increased it (Figure 5). DSE × watering regimen significantly affected the root biomass, total biomass, and root:shoot ratio (Table 2).

### Effects of DSE on Active Ingredient Contents

**FIGURE 6:**
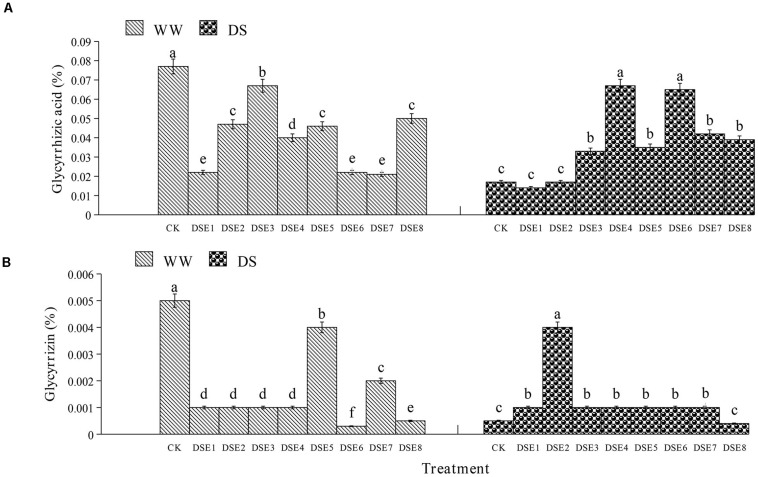
**(A,B)** Effects of dark septate endophytes (DSE) and water treatment on the active ingredient content of licorice plants. Different letters above the error bars indicate significant difference at *P* < 0.05. CK indicates non-inoculated plants. DSE1–DSE8, indicate plants inoculated with different DSE species. WW, DS, indicate well-watered and drought stress treatment, respectively.

Regarding the glycyrrhizic acid content in the roots, DSE colonization had significant effects relative to the control plants, regardless of the watering regime (Figure 6). Under WW conditions, all DSE reduced the glycyrrhizic acid content, relative to the control plants; under DS conditions, DSE3, DSE4, DSE5, DSE6, DSE7, and DSE8 increased its content, while DSE1 and DSE2 had no significant effects (Figure 6). Under WW conditions, all DSE reduced the glycyrrhizin content, relative to the control plants; under DS conditions, DSE1, DSE2, DSE3, DSE4, DSE5, DSE6, and DSE7 increased it (Figure 6). DSE × watering regimen significantly affected the glycyrrhizic acid content (Table 2).

### Effects of DSE on Soil Parameters

**FIGURE 7:**
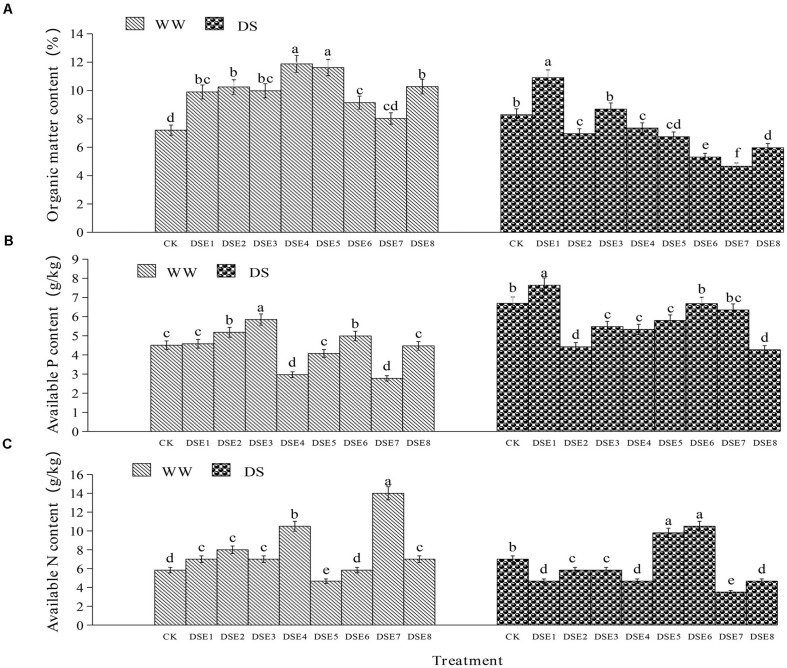
**(A–C)** Effects of dark septate endophytes (DSE) and water treatment on soil parameters. Different letters above the error bars indicate significant difference at *P* < 0.05. CK indicates non-inoculated plants. DSE1–DSE8, indicate plants inoculated with different DSE species. WW, DS, indicate well-watered and drought stress treatment, respectively.

Regarding the soil parameters, DSE colonization had significant effects relative to the control plants, regardless of the watering regimen (Figure 7). Under WW conditions, DSE1, DSE2, DSE3, DSE4, DSE5, DSE6, and DSE8 increased the organic matter content in the rhizopsheric soil, relative to the control plants; DSE2, DSE3, and DSE6 increased the available P, whereas DSE4 and DSE7 decreased it; DSE1, DSE2, DSE3, DSE4, DSE7, and DSE8 increased soil available N, while DSE5 decreased it. Under DS conditions, all DSE increased soil organic matter, relative to the control plants; only DSE1 increased soil available P, while DSE2, DSE3, DSE4, DSE5, and DSE8 reduced it; DSE5 and DSE6 increased soil available N, while DSE1, DSE2, DSE3, DSE4, DSE7, and DSE8 decreased it (Figure 7). DSE × watering regimen significantly affected soil organic matter and available N (Table 3).

**TABLE 3:** Two-way analysis of variance for the effects of dark septate endophytes (DSE) and water condition on soil parameters in the rhizosphere of licorice plants.

	Soil organic matter (%)		Soil available P (g/kg)		Soil available N (g/kg)	
	F	P	F	P	F	P
DSE	1.536	0.169	0.96	0.482	0.965	0.483
Water	14.981	**<0.001**	4.396	**0.042**	6.017	**0.019**
DSE × water	2.437	**0.032**	1.183	0.336	5.575	**<0.001**

*Significant P-values are in bold.*

### Correlation Analyses

**FIGURE 8:**
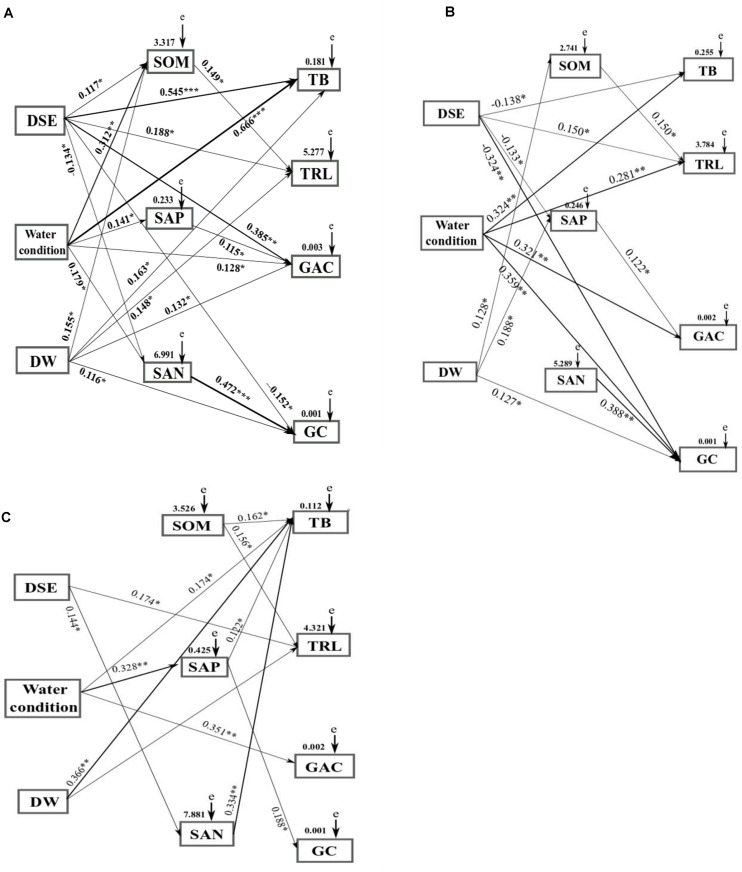
Structural equation model showing the causal relationships among DSE from different sites, water condition, DSE × water, soil parameters, and growth indicators and active ingredients. The final model fitted the data well: maximum likelihood, Shapotou site **(A)** X^2^ = 43.513, *df* = 10, *P* = 0.014, root mean square error of approximation = 0.253, goodness-of-fit index = 0.613, Akaike information criteria = 148.468; Minqin site **(B)** X^2^ = 69.482, *df* = 12, *P* = 0.005, root mean square error of approximation = 0.267, goodness-of-fit index = 0.0.571, Akaike information criteria = 161.992; Anxi site **(C)** X^2^ = 82.476, *df* = 13, *P* = 0.001, root mean square error of approximation = 0.323, goodness-of-fit index = 0.538, Akaike information criteria = 172.965. Solid lines and dashed lines indicate significant and non-significant pathways, respectively. The width of the solid lines indicates the strength of the causal effect, and the numbers near the arrows indicate the standardized path coefficients (^∗^*P* < 0.05, ^∗∗^*P* < 0.01, and ^∗∗∗^*P* < 0.001). DW, combination of DSE and water; SOM, soil organic matter; SAP, soil available P; SAN, soil available N; e, the values of residuals; TB, total biomass; TRL, root length; GAC, glycyrrhizinic acid content; GC, glycyrrhizin content.

To assess the impacts of DSE from different sites, watering regime, DSE × water, and soil parameters on the plant characteristics (root length, biomass, and active component content), a SEM model and the Mantel test were conducted. The Mantel test revealed notable correlations among DSE, watering regime, root length, root biomass, total biomass, glycyrrhizin, glycyrrhizic acid, and soil components (Supplementary Tables 2–4). Combined with the correlation coefficients (*R*-values), a SEM model was made to evaluate the relationship between DSE and all measured parameters for each of the studied sites. From this analysis, it emerged that DSE positively affected the root length, total biomass, glycyrrhizic acid, and soil organic matter, and negatively affected glycyrrhizin and soil available N (Shapotou site) (Figure 8A); and DSE positively affected the root length, whereas adversely affected total biomass, glycyrrhizin, and soil available P (Minqin site) (Figure 8B); and DSE positively affected root length and soil available N (Anxi site) (Figure 8C). Watering regime commonly positively affected the total biomass and glycyrrhizic acid; positively affected soil parameters (Shapotou site); and the root length and glycyrrhizin (Minqin site); and soil available P (Anxi site). DSE × watering regimen showed positive effects on the root length, total biomass, glycyrrhizin, glycyrrhizic acid, and soil organic matter (Shapotou site); and positive effects on glycyrrhizin, soil organic matter, and available P (Minqin site); and positive effects on the total biomass (Anxi site). Soil organic matter positively effected the root length, and soil available P positively affected glycyrrhizic acid, and soil available N positively affected the glycyrrhizin (Shapotou or Minqin site) (Figures 8A,B); soil organic matter positively affected the root length and total biomass, and soil available P positively affected the total biomass and glycyrrhizin, and soil available N positively affected the total biomass (Anxi site) (Figure 8C).

### Variation Partitioning Analysis

**FIGURE 9:**
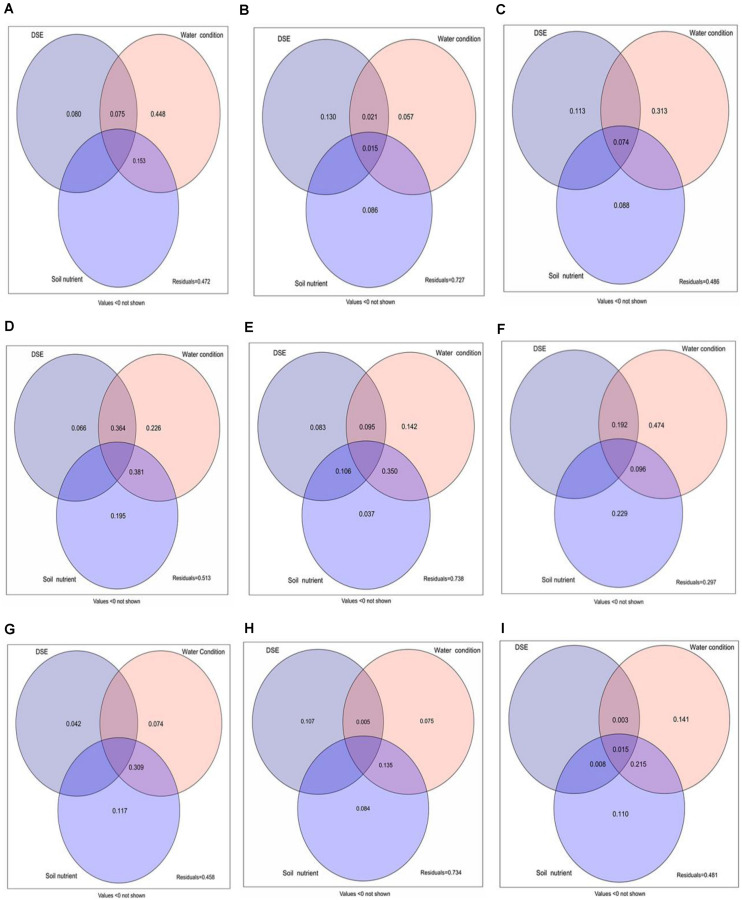
Variation partitioning of DSE from different sites, water condition, and soil parameters on the total biomass **(A,D,G)**, root biomass **(B,E,H)**, and active ingredient contents **(C,F,I)** of licorice plants. DSE, DSE species. Soil nutrient, nutrient content in soil (including soil organic matter, available N, and available P). Values below 0 are not shown. **(A–C)** DSE from Shapotou; **(D–F)** DSE from Minqin; **(G–I)** DSE from Anxi.

Variation partitioning analysis was used to evaluate the contributions of DSE from different sites, watering regime, and soil parameters to the total biomass, root biomass, and active component content (Figure 9). This analysis showed that DSE from Shapotou significantly affected the root biomass and active component content, while watering regimen might be a key factor influencing the total biomass and active component content (Figures 9A–C). DSE from Minqin had a weaker effect on the total biomass, root biomass, and active component content, and soil parameters mainly affected the total biomass and active component content, while watering regimen might be the main factor affecting the total biomass, root biomass, and active component content (Figures 9D–F). DSE from Anxi mainly affected the root biomass, and watering regimen and soil parameters overall affected the total biomass, root biomass, and active component content (Figures 9G–I). Moreover, the analysis showed that the combination of DSE, watering regimen, and soil parameters might be the main contributor to the total biomass and active component content (Figures 9A–I).

## Discussion

### Identification of the DSE Species

In the present study, we found that typical DSE structures, such as dark septate hyphae and microsclerotia, colonized the licorice roots, with a mean colonization rate of 67.3%. The dark septate hyphae of DSE are important for host survival in stressful environments, mainly because oxygen radicals can be trapped and eliminated by melanin in the cell walls during abiotic stress (Yu et al., 2015). In addition, the chlamydospore-like microsclerotia observed in licorice roots may be related to the higher drought stress tolerance among plants (Xie et al., 2017; Hou et al., 2019). Based on morphological and molecular identification, we identified the eight DSE species isolated from licorice roots collected from pedo-climatically different locations as *A. nepalense* (Hypocreales), *A. vagum*, *A. chartarum*, *A. chlamydospora*, *A. longissima*, *D. alpha*, *P. chrysanthemicola*, and *P. terricola* (Pleosporales). Among these DSE species, the last three have been reported as DSE species in desert habitats (Massimo et al., 2015; Li et al., 2018). Regarding the first five DSE species, this is the first report of these species in desert areas in northwest China.

In previous studies, *D. alpha*, a common member of the core DSE community distributed in semiarid grasslands worldwide, was proposed to play a key role as a decomposer of dead roots due to the general increase in carbohydrate active enzymes in DSE fungi (Knapp et al., 2018). The pathogen *P. chrysanthemicola* has been shown to cause root and basal rot, resulting in wilting seedlings (Hay et al., 2015). *Preussia* spp. such as *P. terricola* have been reported to improve plant growth and produce phytohormones (Al-Hosni et al., 2018). *A. nepalense*, a manganese oxide-depositing fungus, can convert soluble Mn (II) into insoluble Mn (III, IV) oxides (Saiz-Jimenez et al., 2012). *A. vagum* from field-grown licorice has been shown to reduce heavy metal content in tobacco leaves (Jin et al., 2018), and to improve the growth and active component content of licorice plants (He et al., 2019a). *Alternaria* spp. are known to be highly pathogenic to plants, causing great losses to many crops (Woudenberg et al., 2013).

### Dark Septate Endophytes Biochemical Characterization

The different DSE species exhibited very diverse growth in response to osmotic stress and to the level of the stress. In general, the cells of stressed organisms produce reactive oxygen species (ROS), and the elevated ROS levels may lead to severe oxidative damage to the cellular biomolecules (Hernández-Pérez et al., 2021). To counteract such damage, the organisms grew more as a part of their defense mechanism (Baxter et al., 2014). Previous research reported that DSE from wild rice grew more at –0.8 MPa relative to 0 MPa (dos Santos et al., 2017). Li et al. (2019a) reported that the intermediate stress conditions (–1.34 or –2.24 MPa) were more suitable, relative to other osmotic stresses, for the growth of DSE obtained from *Hedysarum scoparium*. We conjecture that the osmotic stress tolerance of DSE may be related to the DSE species and their ecological adaptability (He et al., 2019b; Li et al., 2019a).

Osmotic stress usually negatively affects organisms and causes oxidative damage to cells (Liu et al., 2010). In this study, we determined the SOD activity and MDA and melanin content in the eight DSE to assess antioxidant responses to osmotic stress. SOD is one of the most important enzymes for the removal of reactive oxygen species (ROS) (Hosseini et al., 2019). The SOD activity in DSE1, DSE5, DSE7, and DSE8 increased under specific osmotic stress conditions, indicating that SOD was synthesized to remove ROS under intensified stress. In contrast, the reduced SOD activity in DSE2 and DSE3 at –1.34 and –1.79 MPa indicated that other components might contribute to the DSE response to enhanced osmotic stress. In addition, as a biomarker of environmental stress, the MDA content of DSE5 and DSE8 decreased at –1.79 MPA, which indicated that these DSE could resist the detrimental effects of drought stress (Khan et al., 2012; Xu et al., 2017). In contrast, the increased MDA content in DSE3, DSE4, and DSE7 under various osmotic stress conditions implies that osmotic stress enhanced the membrane lipid peroxidation in DSE, as these DSE had decreased tolerance to osmotic stress (Suleiman et al., 2019).

The effect of osmotic stress on the melanin content of DSE depended on the DSE species. Melanin, an antioxidant agent that reduces oxidative damage, protects organisms from environmental stress (Zhan et al., 2011; Liu et al., 2018; Hosseini et al., 2019). Eisenman et al. (2020) also reported that melanin protects fungi from a range of stresses in the environment.

### Effects of DSE Inoculation on Plant and Rhizospheric Soil

Hyphae and microsclerotia were detected in licorice roots inoculated with the eight DSE under drought stress, which indicated that the eight DSE are effective root colonizers even under drought stress. In addition, the response of licorice plants to DSE colonization was strain-dependent. Specifically, licorice plants inoculated with DSE5 had significantly increased root and total biomass compared to the control plants, whereas DSE4 had significant negative effects on the root and total biomass, regardless of the watering regimen. Our observations are consistent with those of previous studies showing that the DSE species influences the DSE–plant interaction (He et al., 2019b; Li et al., 2019a, b). The SEM analysis showed that DSE from various desert habitats directly affected the root length, active component content of roots, and plant biomass. This study is the first to report the neutral and positive effects of *Alternaria* (i.e., *Alternaria chartarum*, DSE8), which is considered a plant pathogen (Woudenberg et al., 2013). Moreover, although no DSE except DSE5 affected the shoot biomass, DSE improved the root biomass under drought stress. Thus, our results suggested that DSE promoted the growth of licorice plants under drought stress, potentially by altering the osmotic stress tolerance or the nutrient solubilization capacity regarding the rhizospheric soil (Porras-Alfaro et al., 2008; Li et al., 2018). Furthermore, our results revealed that DSE × watering regimen significantly affected the glycyrrhizic acid content in the host plants. These findings suggested that DSE colonization could alleviate the negative effects of drought stress on the active component content and plant growth (He et al., 2019b).

Both the DSE species and watering regimen affected the root morphological traits. Specifically, DSE1, DSE2, DSE4, DSE5, DSE7, and DSE8 positively affected the root surface area under drought stress, whereas DSE6 negatively affected it under drought stress. Enhanced root growth (e.g., root biomass, length, and surface area) is beneficial for plant roots as it allows them to extend into deeper soil layers and improve water extraction from the soil, thereby increasing plant survival and resistance to ground evaporation in desert ecosystems (Schulze et al., 1996).

Soil organic matter and available N in the rhizospheric soil were positively affected by DSE × watering regimen. The SEM analysis and variation partitioning also indicated that DSE directly influenced soil nutrient properties. As a bridge between the plant and soil environment, DSE are a key element of plant nutrient uptake; their underground mycelium network facilitates nutrient transfer from the soil to colonized roots (Usuki and Narisawa, 2007; Alberton et al., 2010; Vergara et al., 2019). For example, there were some positive correlations between a specific DSE and the total biomass and root length in this study. Barrow (2003) also suggested that septate endophytic hyphae improved nutrient and water transport by extending > 300 μm from the root matrix during extended DS. Additionally, DSE can play important roles in host nutrition via complex substrate degradation mechanisms; secreted enzymes from DSE can degrade organic matter and mineralize organic N and insoluble P into available forms; thus, plant growth and tolerance can be promoted under stress (Caldwell et al., 2000; Surono and Narisawa, 2017; He et al., 2019a).

Some studies have found that different DSE and DSE–plant interactions respond differently to abiotic stresses (Elvira-Recuenco et al., 2014; Kia et al., 2017; Bragulat et al., 2019). For instance, under drought stress, *A. vagum* from cultivated licorice in arid farmland increased the total biomass, root biomass, and glycyrrhizic acid content of the licorice plant (He et al., 2019b). In the current study, under drought stress, the total biomass and root biomass were reduced, and the glycyrrhizin content was increased, for licorice plants inoculated with *A. vagum* from wild licorice in a desert environment. These findings regarding the different effects of DSE isolated from different habitats on plant growth and compound production confirm the specific nature of DSE–plant interactions, as proposed by other authors (Mandyam and Jumpponen, 2005; Li et al., 2019a). To our knowledge, the increase in licorice plant growth and drought stress tolerance might be attributable to improved soil nutrition, microbiota, and root structure (González-Teuber et al., 2018; Changey et al., 2019; He et al., 2019b). According to the variation partitioning analysis, there were certain degrees of unexplained variations in the total biomass, root biomass, and active component content, which indicates that unexplored factors (such as the DSE inoculation volume and degree of drought stress) affect licorice plant growth and active component accumulation.

## Conclusion

In the present study, we first characterized eight DSE species from wild licorice plants in desert habitats and investigated the performance of licorice plants after DSE inoculation under drought stress. The osmotic stress tolerance of the eight DSE species was highly variable and there were obvious functional differences in the performance of inoculated licorice plants. The response of licorice plants to DSE varied from neutral to beneficial depending on both the DSE species and watering regimen. Specifically, *A. chlamydospora* and *P. terricola* increased the total biomass and root biomass of licorice plants under drought stress; all DSE except *A. vagum* and *P. chrysanthemicola* increased the glycyrrhizic acid content under drought stress; and all DSE except *A. chartarum* increased the glycyrrhizin content under drought stress; Additionally, DSE × watering regimen increased the root glycyrrhizic acid content and the available N and organic matter in the rhizospheric soil. Furthermore, the DSE–plant interaction was affected by DSE species and DSE originating habitat. As *A. chlamydospora* and *P. terricola* positively affected the total biomass, root biomass, and active component content under drought stress, they may have important uses as promoters in the cultivation of licorice plants in arid areas.

## Data Availability Statement

The datasets generated for this study can be found in the sequences were stored in GenBank with accession numbers of MN517851 (DSE1), MN517852 (DSE2), MN517853 (DSE3), MN517854 (DSE4), MN517855 (DSE5), MN517856 (DSE6), MN517857 (DSE7), and MN517858 (DSE8).

## Author Contributions

CH and WW conceived and designed the experiments. CH and JH performed the experiments. CH, JH, and XL analyzed the data. CH, WW, and XL wrote the manuscript. All authors contributed to the article and approved the submitted version.

## Conflict of Interest

The authors declare that the research was conducted in the absence of any commercial or financial relationships that could be construed as a potential conflict of interest.
