# Supplementary material for: Dark Septate Endophytes Isolated From Wild Licorice Roots Grown in the Desert Regions of Northwest China Enhance the Growth of Host Plants Under Water Deficit Stress
Source: Front Microbiol. 2021 Jun 23;12:522449. doi: 10.3389/fmicb.2021.522449 (PMC8260703; doi:10.3389/fmicb.2021.522449)
Supplement: Supplementary file 1 [file Data_Sheet_1.doc]

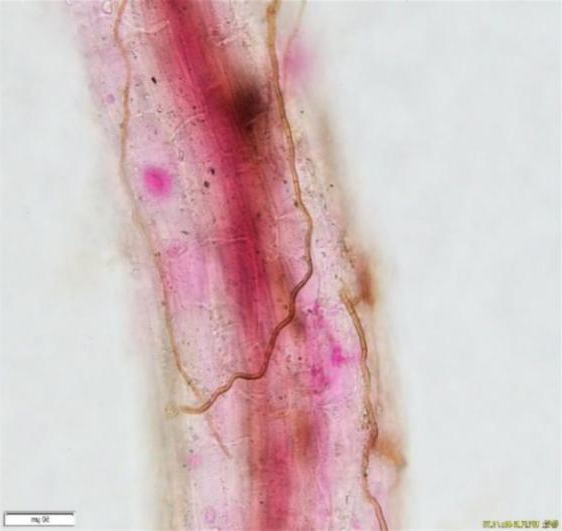


**A**

**50μm**

**Hy**


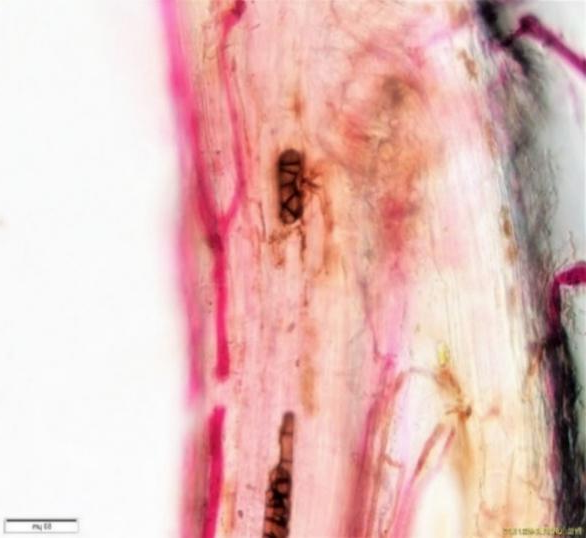


**B**

**50μm**

**M**


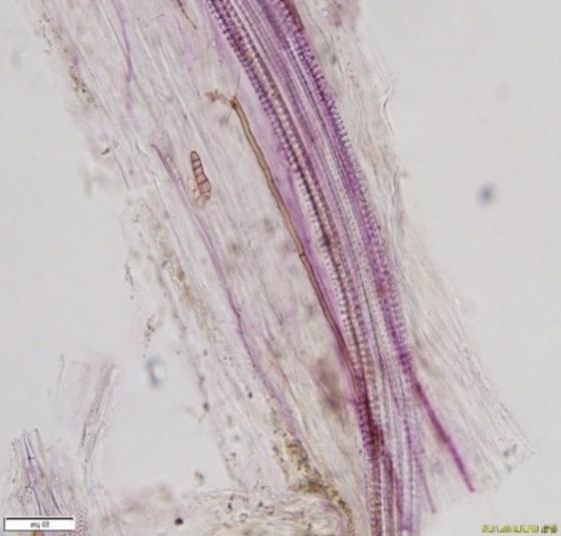


**C**

**50μm**

**M**

**Hy**


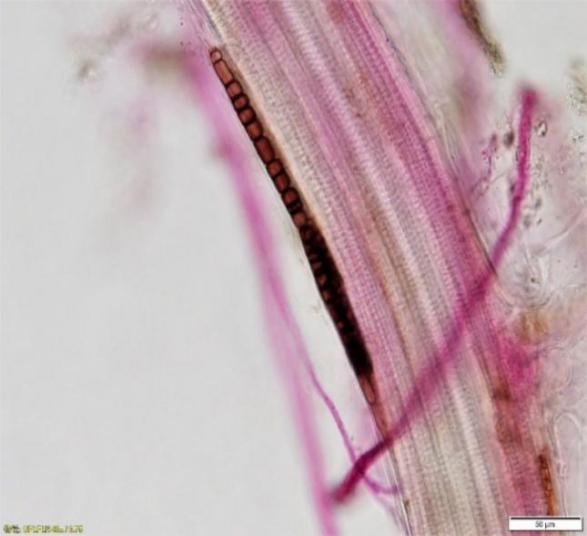


**D**

**50μm**

**M**


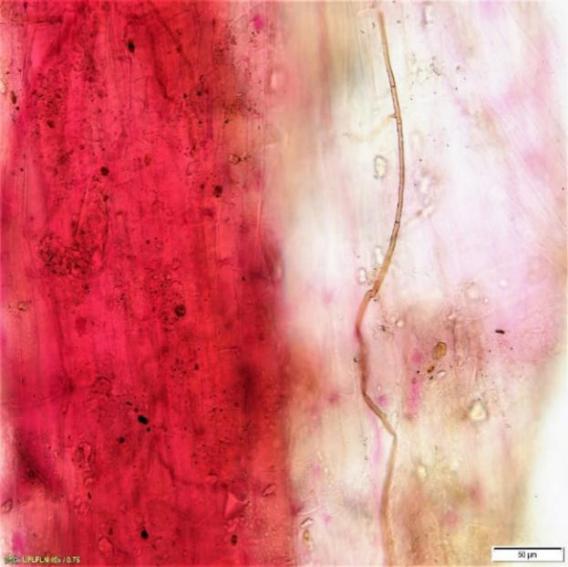


**E**

**50μm**

**Hy**


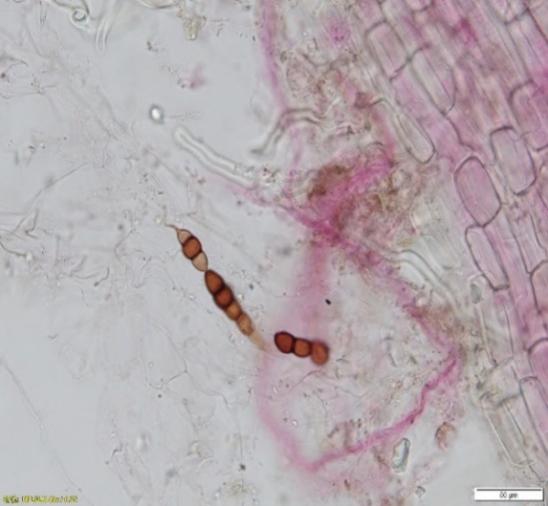


**F**

**50μm**

**M**

**Figure S1** Colonization of dark septate endophyte (DSE) strains in the roots of wild

licorice grown in different desert sites. A and B indicate root samples from Shapotou; C and D indicate root samples from Minqin; E and F indicate root samples from Anxi.

Arrows indicate: Hy=DSE hyphae; M=DSE microsclerotia.


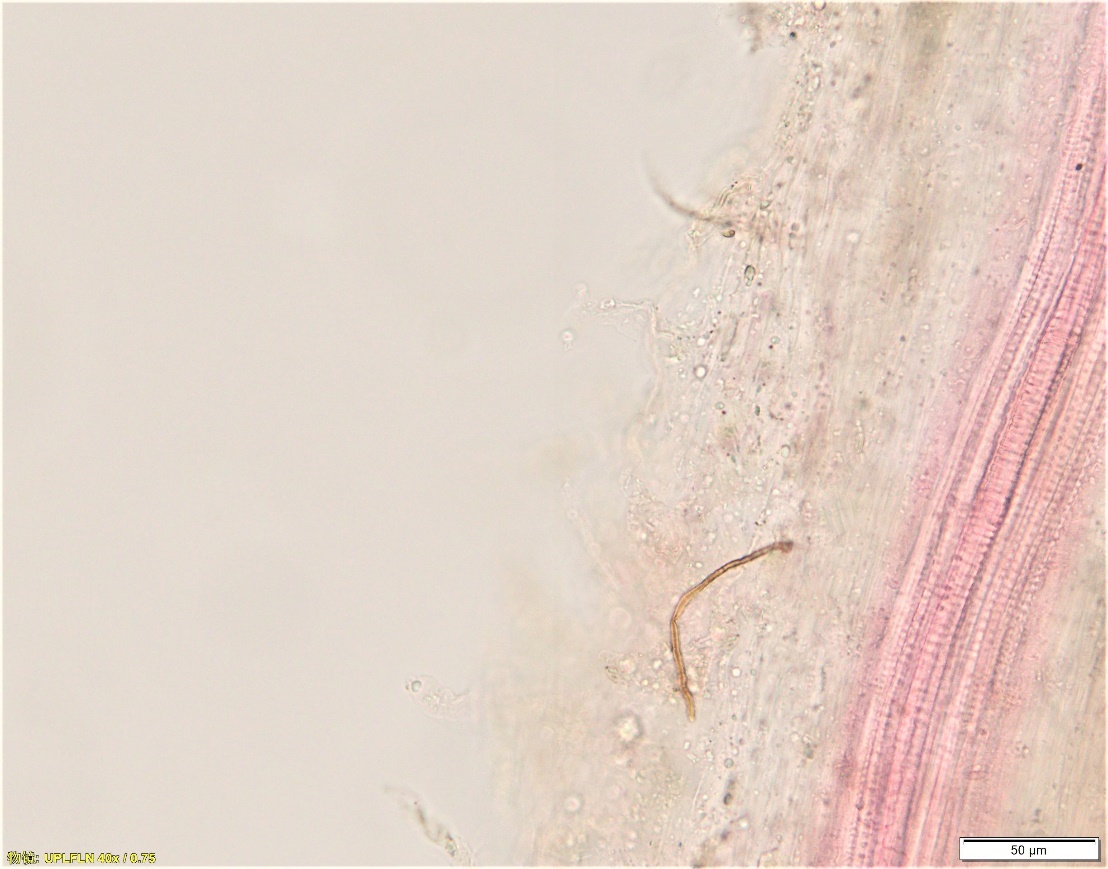


**E**

**Hy**

**50μm**


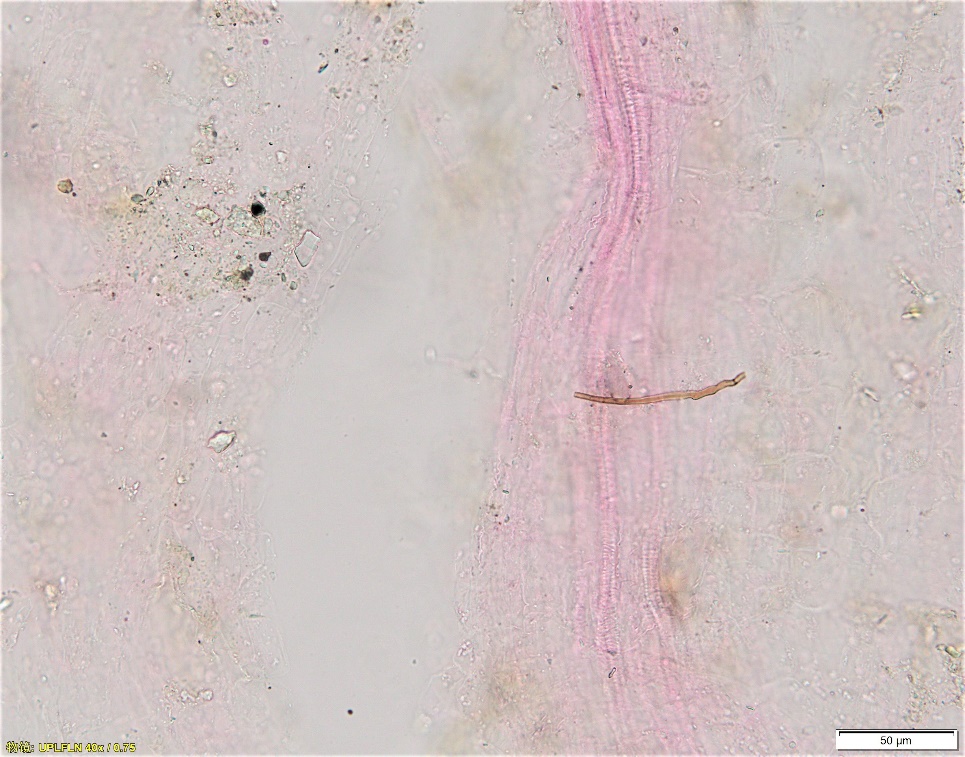


**C**

**Hy**

**50μm**


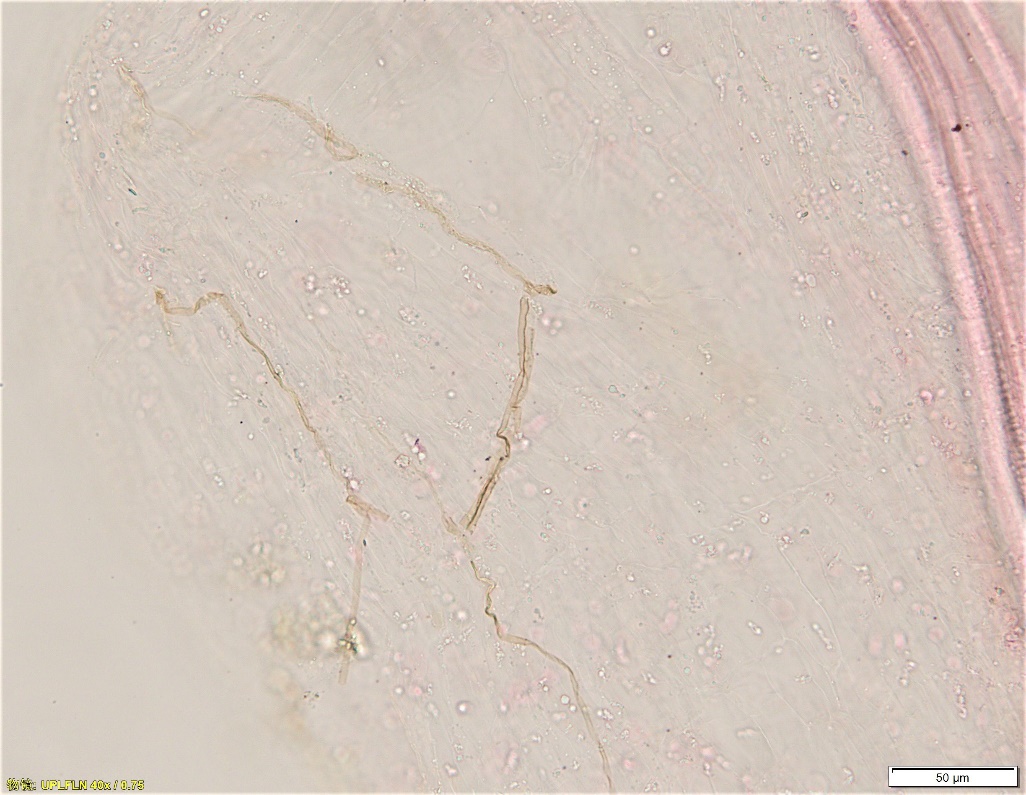


**A**

**50μm**

**Hy**


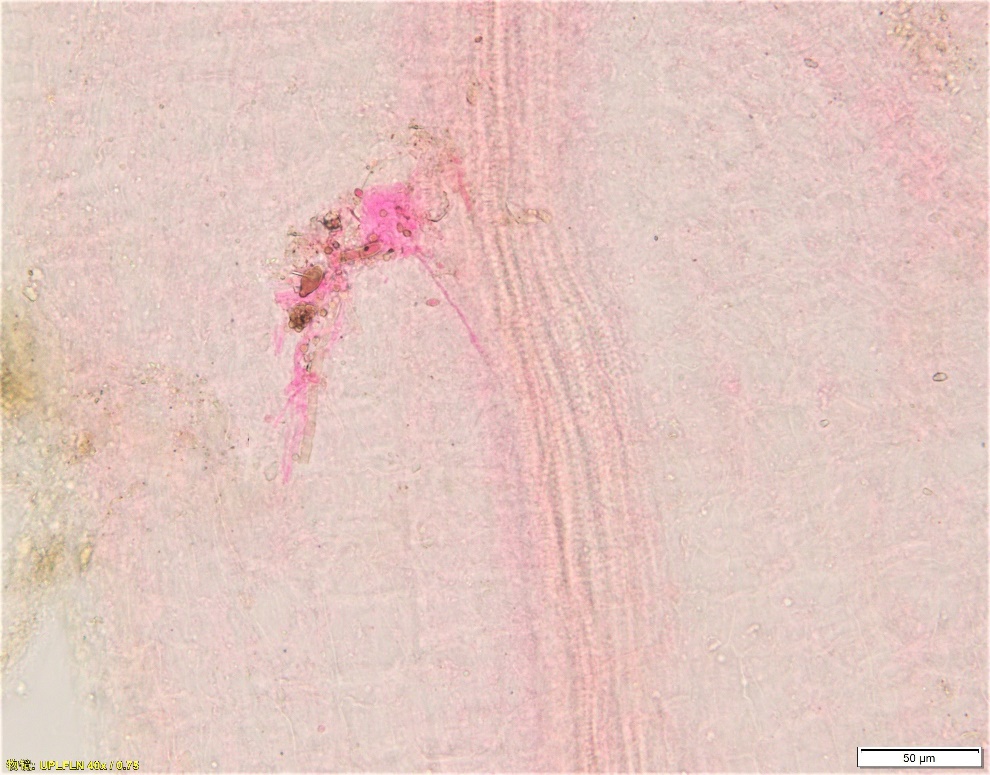


**B**

**50μm**

**M**


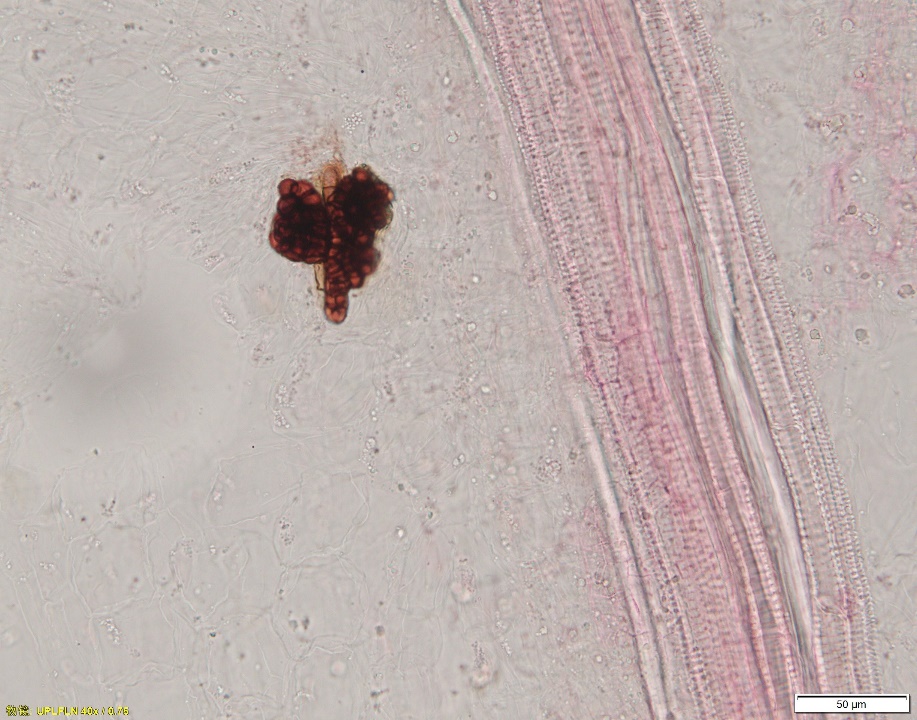


**D**

**50μm**

**M**


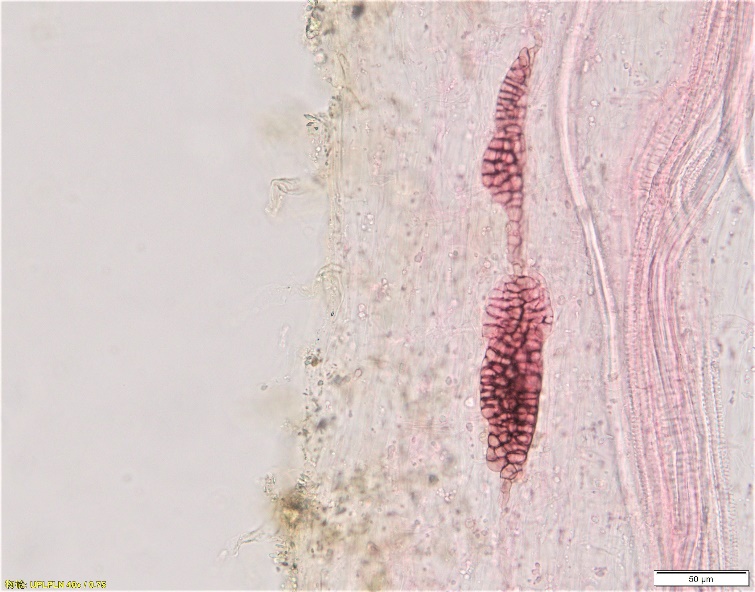


**F**

**50μm**

**M**


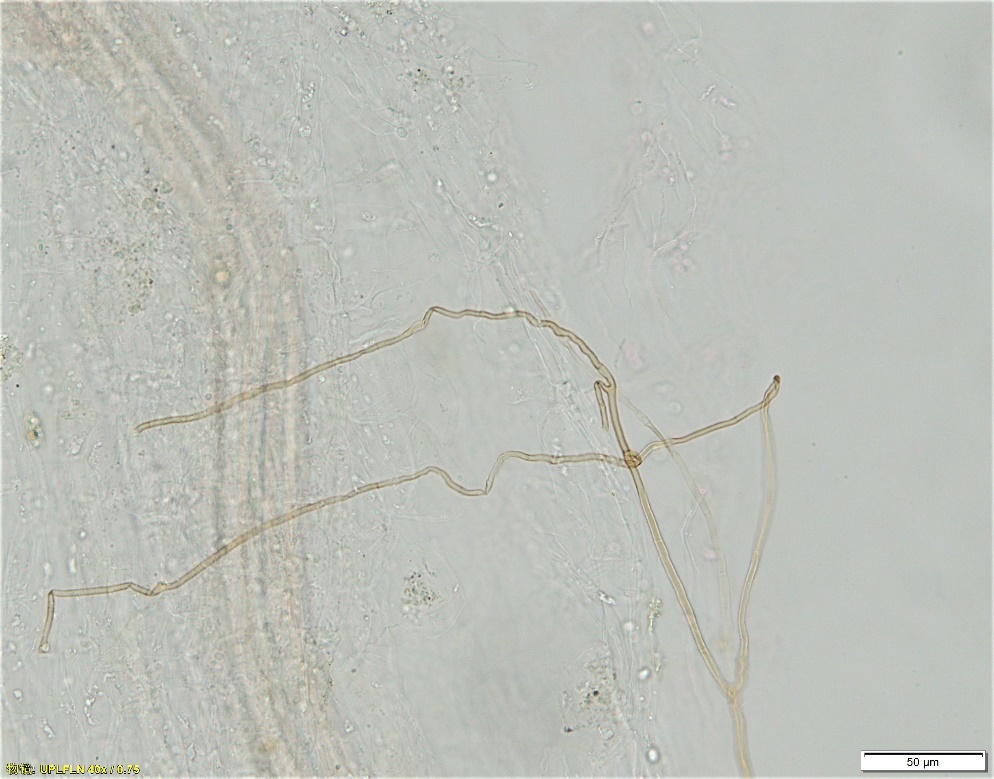


**G**

**50μm**

**Hy**


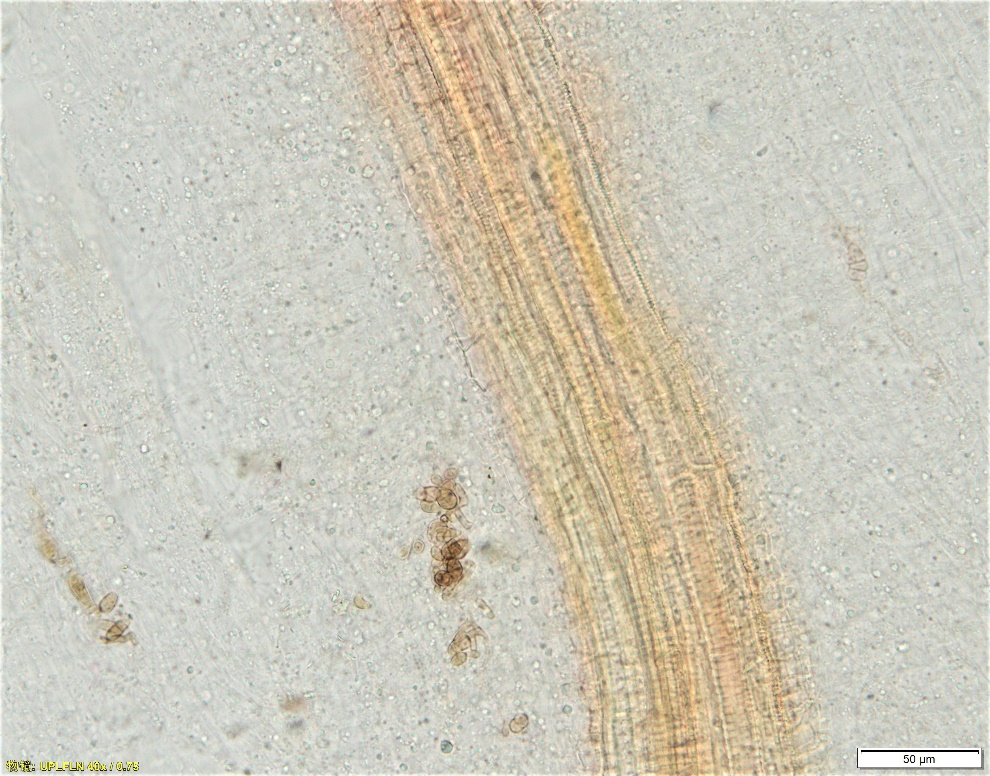


**H**

**50μm**

**M**


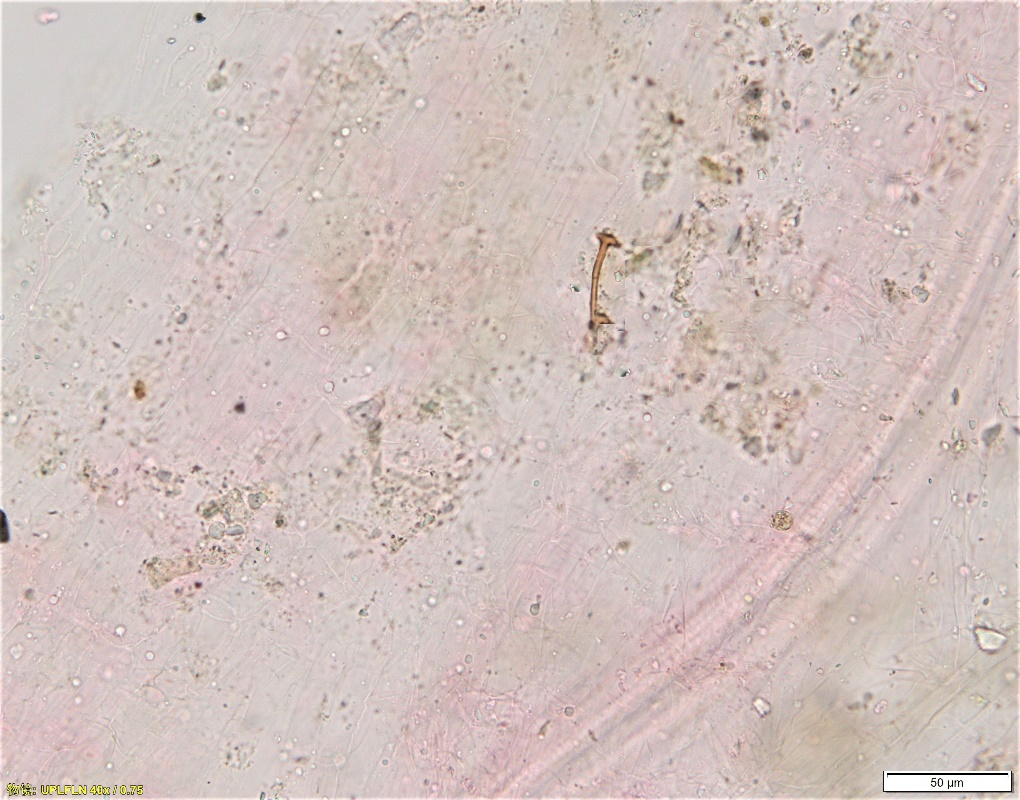


**I**

**50μm**

**Hy**


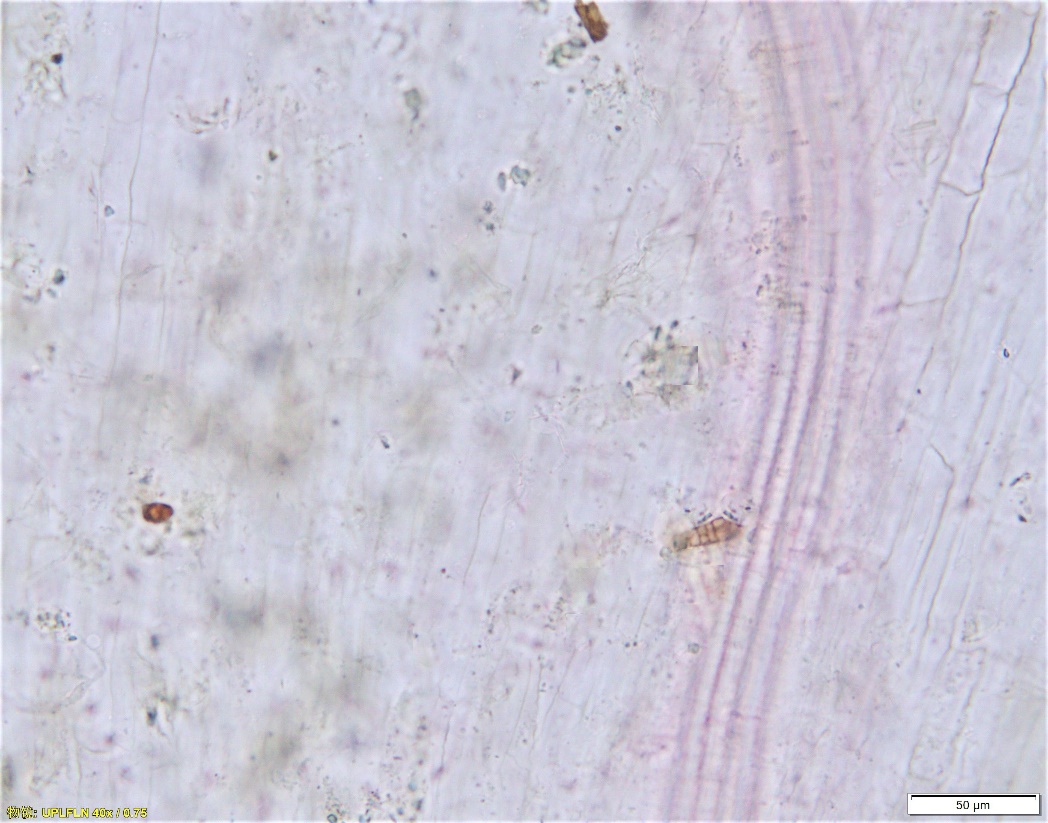


**J**

**50μm**

**M**


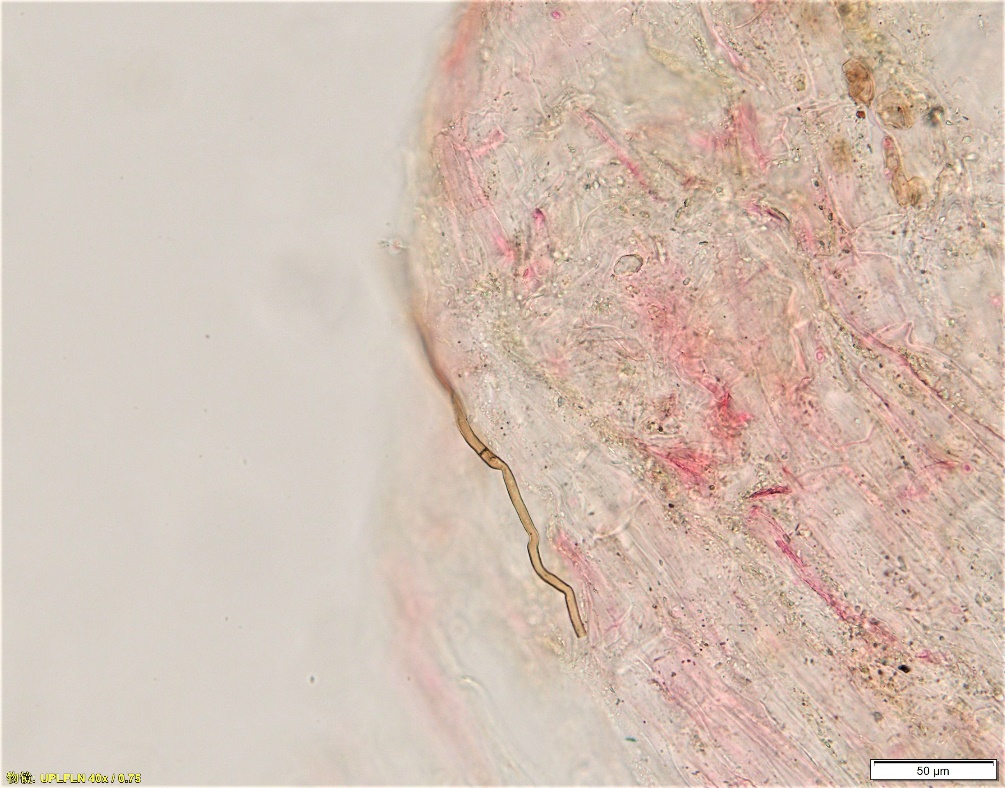


**K**

**50μm**

**Hy**


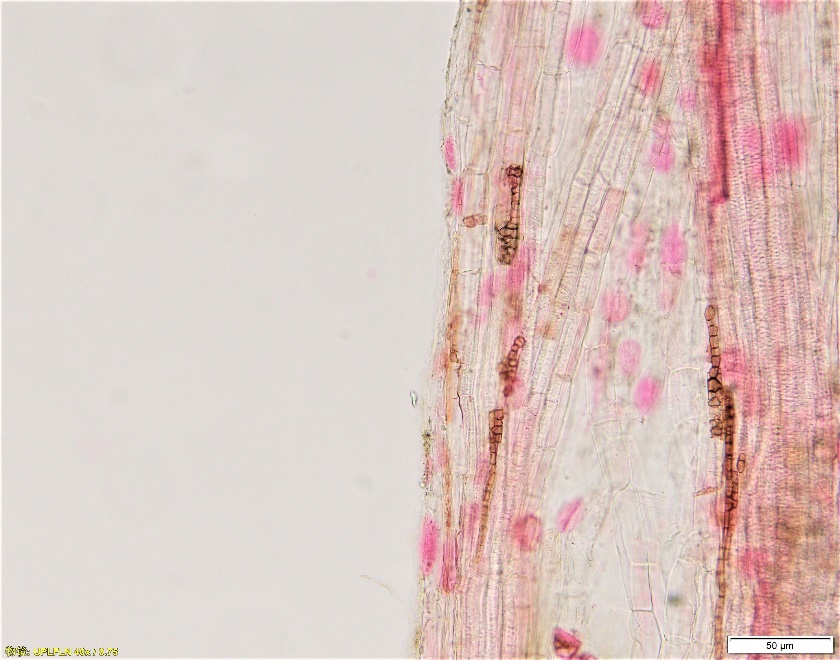


**L**

**50μm**

**M**


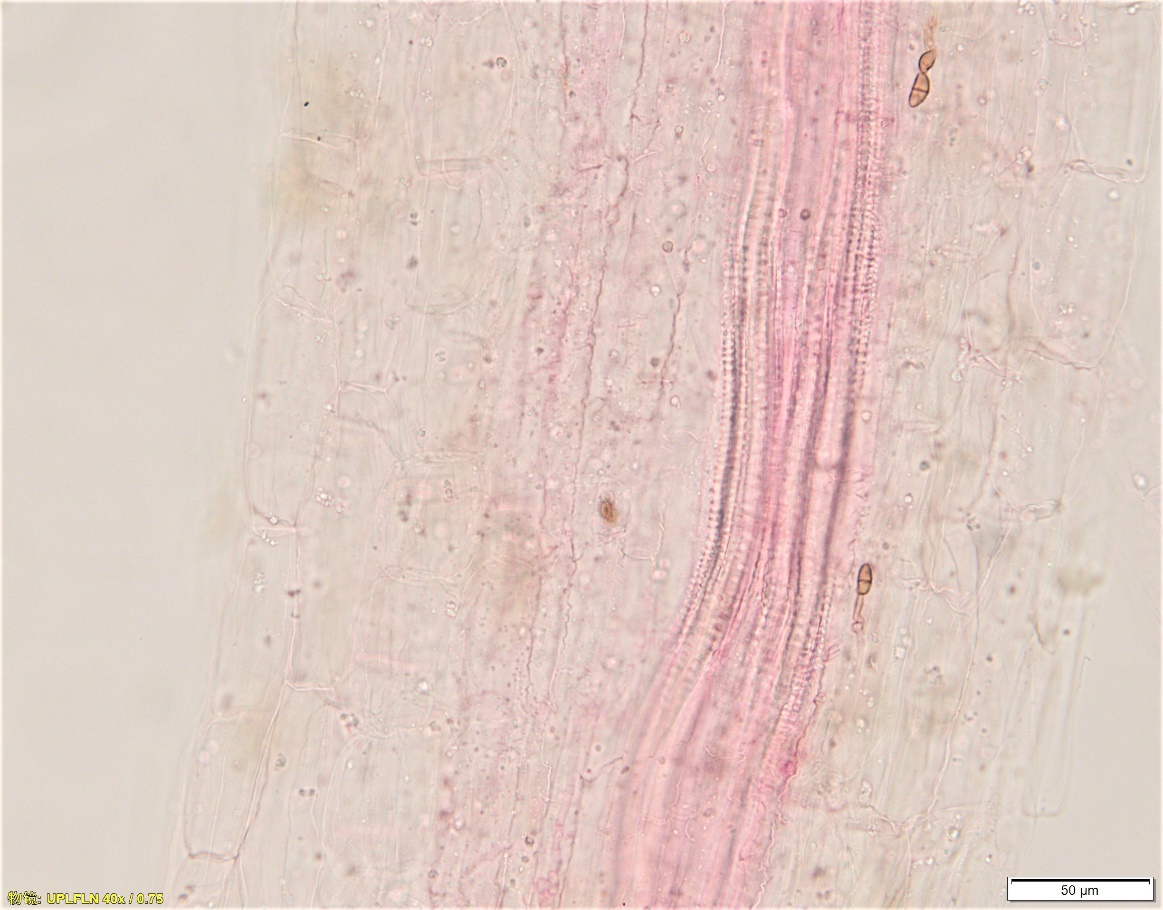


**P**

**50μm**

**M**


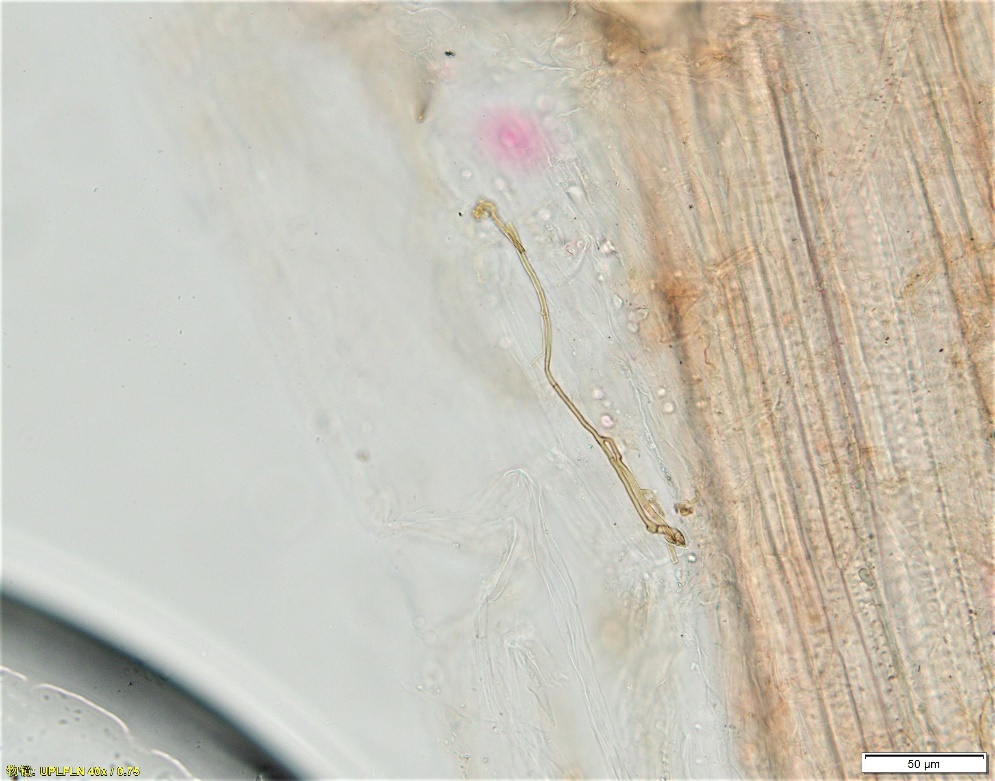


**O**

**50μm**

**Hy**


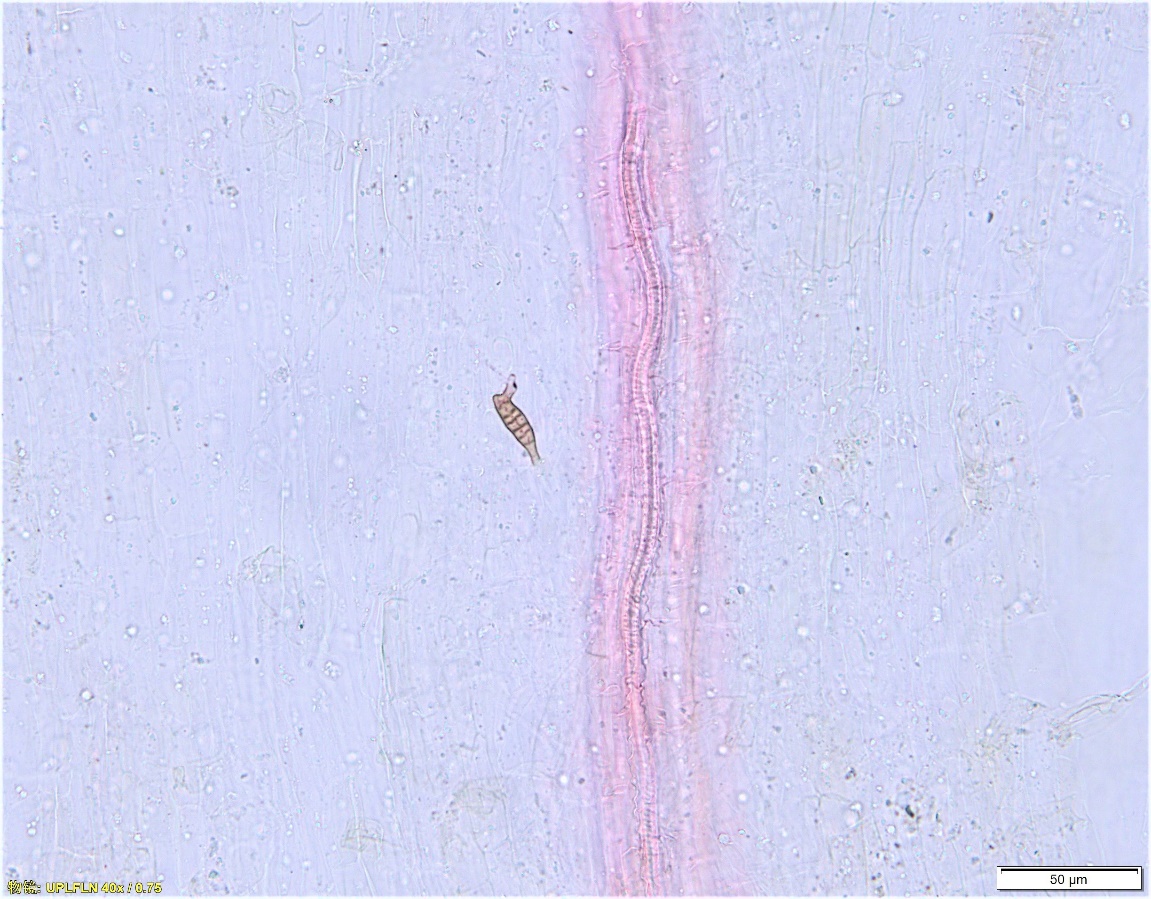


**N**

**50μm**

**M**


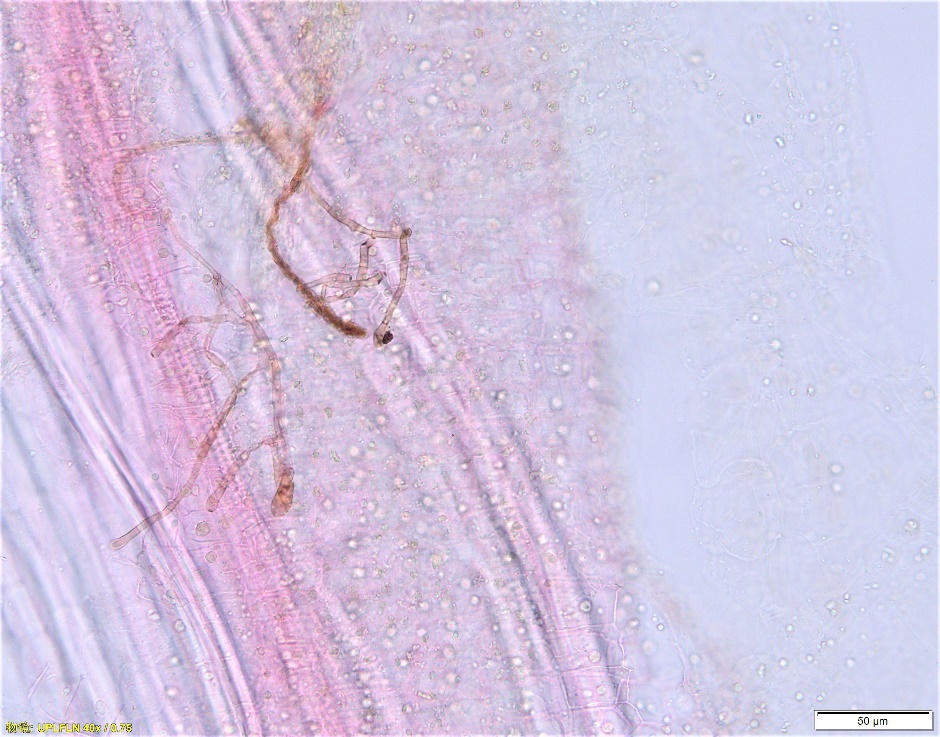


**M**

**50μm**

**Hy**

**Figure S2** Colonization of dark septate endophyte (DSE) strains in the roots of licorice plant three months after inoculation. A, B: inoculation with DSE1; C, D:inoculation with DSE2; E, F: inoculation with DSE3; G, H: inoculation with DSE4; I, J: inoculation with DSE5; K, L: inoculation with DSE6; M, N: inoculation with DSE7; O, P: inoculation with DSE8; respectively. Arrows indicate: Hy=DSE hyphae; M=DSE microsclerotia.

**Table S1 HPLC mobile phase and** [**gradient elution**](http://dict.cnki.net/dict_result.aspx?searchword=梯度洗脱条件&tjType=sentence&style=&t=condition+of+gradient+elution)**.**

| Time (min) | A (acetonitrile) | B (deionized water : phosphoric acid) |
| --- | --- | --- |
| 0.0 | 14% | 86% |
| 10.0 | 23% | 77% |
| 24.0 | 30% | 70% |
| 30.0 | 34% | 66% |
| 35.0 | 36% | 64% |
| 42.0 | 42% | 58% |
| 48.0 | 51% | 49% |
| 60.0 | 14% | 86% |

**Table S2** Mantel tests showing correlationships (R values) between DSE from Shapotou, water condition, soil organic matter, soil available P, soil available N, total biomass, total root length, glycyrrhizic acid and glycyrrhizin of licorice plants.

| Variable | DSE | Water | DW | SOM | SAP | SAN | TB | TRL | GAC | GC |
| --- | --- | --- | --- | --- | --- | --- | --- | --- | --- | --- |
| DSE | 1 |  |  |  |  |  |  |  |  |  |
| Water | -0.048 | 1 |  |  |  |  |  |  |  |  |
| DW | 0.502*** | -0.024 | 1 |  |  |  |  |  |  |  |
| SOM | 0.204* | 0.159* | 0.161* | 1 |  |  |  |  |  |  |
| SAP | 0.014 | 0.188* | -0.033 | 0.008 | 1 |  |  |  |  |  |
| SAN | -0.198* | 0.232* | -0.068 | 0.235* | 0.012 | 1 |  |  |  |  |
| TB | 0.428*** | 0.637*** | 0.215* | -0.048 | -0.104 | -0.020 | 1 |  |  |  |
| TRL | 0.155* | -0.081 | 0.166* | 0.200* | 0.062 | -0.073 | 0.158* | 1 |  |  |
| GAC | 0.274** | 0.155* | 0.164* | 0.101 | 0.151* | -0.006 | -0.048 | 0.031 | 1 |  |
| GC | -0.135* | -0.011 | 0.142* | 0.048 | -0.100 | 0.415*** | 0.046 | -0.124 | 0.085 | 1 |

**Table S3** Mantel tests showing correlationships (R values) between DSE from Minqin, water condition, soil organic matter, soil available P, soil available N, total biomass, total root length, glycyrrhizic acid and glycyrrhizin of licorice plants.

| Variable | DSE | Water | DW | SOM | SAP | SAN | TB | TRL | GAC | GC |
| --- | --- | --- | --- | --- | --- | --- | --- | --- | --- | --- |
| DSE | 1 |  |  |  |  |  |  |  |  |  |
| Water | 0.201* | 1 |  |  |  |  |  |  |  |  |
| DW | 0.624*** | -0.005 | 1 |  |  |  |  |  |  |  |
| SOM | 0.034 | 0.101 | 0.144* | 1 |  |  |  |  |  |  |
| SAP | -0.163* | 0.023 | 0.215* | -0.106 | 1 |  |  |  |  |  |
| SAN | -0.045 | -0.045 | -0.045 | -0.077 | -0.055 | 1 |  |  |  |  |
| TB | -0.182* | 0.365** | -0.084 | -0.097 | 0.079 | 0.025 | 1 |  |  |  |
| TRL | 0.134* | 0.327** | -0.099 | 0.169* | 0.083 | -0.079 | 0.123* | 1 |  |  |
| GAC | 0.045 | 0.399*** | 0.045 | -0.088 | 0.144 | -0.041 | 0.432*** | -0.066 | 1 |  |
| GC | -0.411*** | 0.521*** | 0.244** | -0.090 | -0.194 | 0.361** | 0.169* | -0.081 | 0.085 | 1 |

**Table S4** Mantel tests showing correlationships (R values) between DSE from Anxi, water condition, soil organic matter, soil available P, soil available N, total biomass, total root length, glycyrrhizic acid and glycyrrhizin of licorice plants.

| Variable | DSE | Water | DW | SOM | SAP | SAN | TB | TRL | GAC | GC |
| --- | --- | --- | --- | --- | --- | --- | --- | --- | --- | --- |
| DSE | 1 |  |  |  |  |  |  |  |  |  |
| Water | -0.041 | 1 |  |  |  |  |  |  |  |  |
| DW | 0.756*** | -0.047 | 1 |  |  |  |  |  |  |  |
| SOM | -0.066 | -0.071 | -0.069 | 1 |  |  |  |  |  |  |
| SAP | -0.002 | 0.356** | -0.046 | -0.016 | 1 |  |  |  |  |  |
| SAN | 0.177* | -0.155 | -0.055 | -0.155* | 0.216* | 1 |  |  |  |  |
| TB | -0.119 | 0.202* | 0.475*** | 0.192* | 0.166* | 0.422*** | 1 |  |  |  |
| TRL | 0.188* | -0.105 | 0.157* | 0.178* | -0.045 | 0.008 | 0.311** | 1 |  |  |
| GAC | 0.026 | 0.488*** | 0.021 | 0.022 | -0.008 | -0.032 | 0.021** | -0.045 | 1 |  |
| GC | 0.074 | 0.088 | 0.088 | 0.081 | 0.145* | 0.091 | 0.372** | 0.085 | 0.158* | 1 |
